# 1800 MHz Radiofrequency Electromagnetic Field Impairs Neurite Outgrowth Through Inhibiting EPHA5 Signaling

**DOI:** 10.3389/fcell.2021.657623

**Published:** 2021-04-12

**Authors:** Chunhai Chen, Qinglong Ma, Ping Deng, Min Lin, Peng Gao, Mindi He, Yonghui Lu, Huifeng Pi, Zhixin He, Chao Zhou, Yanwen Zhang, Zhengping Yu, Lei Zhang

**Affiliations:** ^1^Department of Occupational Health, Third Military Medical University, Chongqing, China; ^2^Key Laboratory of Electromagnetic Radiation Protection, Ministry of Education, Chongqing, China

**Keywords:** radiofrequency electromagnetic fields, neural stem cells, neuron, neurite outgrowth, EPHA5

## Abstract

The increasing intensity of environmental radiofrequency electromagnetic fields (RF-EMF) has increased public concern about its health effects. Of particular concern are the influences of RF-EMF exposure on the development of the brain. The mechanisms of how RF-EMF acts on the developing brain are not fully understood. Here, based on high-throughput RNA sequencing techniques, we revealed that transcripts related to neurite development were significantly influenced by 1800 MHz RF-EMF exposure during neuronal differentiation. Exposure to RF-EMF remarkably decreased the total length of neurite and the number of branch points in neural stem cells-derived neurons and retinoic acid-induced Neuro-2A cells. The expression of Eph receptors 5 (EPHA5), which is required for neurite outgrowth, was inhibited remarkably after RF-EMF exposure. Enhancing EPHA5 signaling rescued the inhibitory effects of RF-EMF on neurite outgrowth. Besides, we identified that cAMP-response element-binding protein (CREB) and RhoA were critical downstream factors of EPHA5 signaling in mediating the inhibitory effects of RF-EMF on neurite outgrowth. Together, our finding revealed that RF-EMF exposure impaired neurite outgrowth through EPHA5 signaling. This finding explored the effects and key mechanisms of how RF-EMF exposure impaired neurite outgrowth and also provided a new clue to understanding the influences of RF-EMF on brain development.

## Introduction

The popularity, widespread use and increasing dependency on wireless, intelligent communication, and surveillance technologies have increased public exposure to radiofrequency electromagnetic fields (RF-EMF) (Bandara and Carpenter, 2018). Mounting scientific evidence suggests that prolonged RF-EMF exposure from cell phones, base stations, and other electrical devices has hazardous biological and health effects (Miller et al., 2019). The notable effects of RF-EMF exposure include increasing tumor risk, impairing neurodevelopment, and elevating the risk of some neurodegenerative diseases (Zhang et al., 2016). Especially, the influences of RF-EMF exposure on brain development in children have raised great concern. Children have a relatively thin skull’s bone and higher water content in their brain tissue (Kaplan et al., 2016). Thus, the penetration and absorption rate of RF-EMF in children is relatively higher than in adults (Fernandez et al., 2018). Also, given the greater susceptibility of the developing brain to environmental hazards, it is crucial to explore how RF-EMF exposure influences the developing brain.

Population-based studies have revealed that women exposed to RF-EMF during pregnancy have adverse effects on the neurodevelopment of offspring, and increase their odds of emotional and behavioral difficulties (Sudan et al., 2016; Choi et al., 2017). Studies focus on adolescents students exposed to mobile phones or base stations have revealed that RF-EMF exposure is associated with the impairment of spatial memory, attention deficiency, and delayed motor skills (Foerster et al., 2018; Meo et al., 2019). Besides, it has been revealed that RF-EMF exposure causes neural behavior changes in rats and mice, such as influenced emotional behavior, decreased locomotor activities, and impairment of cognitive functions (Zhang et al., 2017; Kim et al., 2019b). RF-EMF exposure also influenced the electrical activity in neuronal networks in the brain and peripheral neurons (El Khoueiry et al., 2018; Prucha et al., 2018). Recently, it has been found that prenatal RF-EMF exposure inhibits the proliferation and differentiation of embryonic neural stem cells (NSCs), which affects the neurological functions of adults (Kaplan et al., 2016; Eghlidospour et al., 2017). Also, impaired neurite outgrowth in cultured cells after RF-EMF exposure has been reported previously (Del Vecchio et al., 2009; Chen et al., 2014; Su et al., 2018). Axon and dendrite growth and architecture is a key process during brain development, which is the fundament of the formation of neuronal networks (Stoeckli, 2018; Lanoue and Cooper, 2019). Since the electrical signal is an important signal for axon and dendrite guidance and growth (McCaig et al., 2009), it indicated that RF-EMF exposure would have significant effects on neurite outgrowth. These investigations emphasized that more studies are needed to reveal the influence of RF-EMF exposure on the growth and branching of the neurite. Particularly, the underlying mechanisms should be addressed to better understand how RF-EMF acts on brain development.

The potential mechanisms that RF-EMF acts on the brain indicated by previous studies including influencing the normal functions of the blood-brain barrier (Sirav and Seyhan, 2016; Poulletier de Gannes et al., 2017), inducing neuroinflammation (Lameth et al., 2017), altering the activity of specific calcium channels (Buckner et al., 2015), inducing autophagy (Kim et al., 2018b), and stimulating oxidative stress (Tsoy et al., 2019). Previous studies have also indicated that RF-EMF exposure changes the expression of specific genes related to neuronal development (Nikolova et al., 2005; Zhao et al., 2007). However, due to insufficient data, still much is unknown for RF-EMF acts on those early stages of brain development, for example, the neuronal differentiation of embryonic NSCs and the process of neurite outgrowth. Thus, it is critical to explore the detailed responses of developing neuronal cells after RF-EMF exposure and to reveal the exact effects and mechanisms of RF-EMF on brain development.

Here, to address these questions, we applied high-throughput RNA sequencing (RNA-seq) techniques to explore the key pathways that changed in NSC-derived cells after 1800 MHz RF-EMF exposure. We also verified the results from RNA-seq and revealed that exposure to 1800 MHz RF-EMF inhibited neurite outgrowth in NSC-derived neurons and retinoic acid (RA)-induced Neuro-2A cells. Also, we explored that Eph receptors 5 (EPHA5), which is indicated by RNA-seq analysis, plays a key role in RF-EMF-induced inhibitory effects on neurite outgrowth. This research revealed critical mechanisms of how RF-EMF exposure impaired neurite outgrowth and also provided new insights on understanding the effects of RF-EFM on the developing brain.

## Materials and Methods

### Cell Culture

The culture of embryonic NSCs was carried out according to previous publications (Hirabayashi et al., 2004; Chen et al., 2014) with some modifications. Briefly, the NSCs were isolated from telencephalons of E11.5 mice and cultured in a mixture medium of Dulbecco’s modified Eagle’s medium (DMEM) and F12 medium (v/v = 1:1, Gibco, Thermo Fisher Scientific, United States). B27 (1×) supplements (Gibco), N2 (1×) supplements (Gibco), fibroblast growth factor-basic (bFGF) (20 ng/mL; Sigma-Aldrich, United States), and epidermal growth factor (EGF) (20 ng/mL; Sigma-Aldrich) were added to the medium before culture. NSCs were cultured under floating conditions to let the cells form neurospheres. The culture medium was half-changed every 3 days. To induce differentiation, poly-L-lysine (Sigma-Aldrich) was used to coat the culture dishes or wells. The formed neurospheres were dissociated with accutase (Gibco) into single cells. The culture density of the cells was adjusted to 1 × 10^5^ cells/ml. EGF and bFGF were removed from the medium, and 1% fetal bovine serum (FBS) (Hyclone, United States) and 1 μM RA (Sigma-Aldrich) was added to the medium. The NSC-derived cells were exposed to RF-EMF after 2 days of differentiation.

For the culture of Neuro-2A cells, a DMEM high glucose medium (Gibco) with 10% FBS was used. RA (Sigma-Aldrich) was used to induce Neuro-2A differentiation at a final concentration of 10 μM, and FBS (Hyclone) was reduced to 1%. The culture cell density was adjusted to 4 × 10^4^ cells/ml. The Neuro-2A cells were exposed to RF-EMF after 24 h of differentiation.

### RF-EMF Exposure

The sXc-1800 exposure system (IT’IS Foundation, Zurich, Switzerland) was used to expose the cells. The details of the exposure system have been described previously (Chen et al., 2014). The exposure chambers contained two 6-dish holders. One received RF-EMF exposure and the other was sham exposure control. The cells were cultured in a monolayer manner when receiving RF-EMF exposure. The temperature in the exposure chambers was monitored and maintained at 37.0 ± 0.5°C. Cells were exposed to a carrier frequency of 1800 MHz RF-EMF in a GSM Talk-signal mode. A 5 min on, 10 min off intermittent exposure mode was used and lasted for 48 h. An average specific absorption ratio (SAR) value of 4 W/kg was selected according to our previous finding (Chen et al., 2014).

### mRNA Sequencing

Neural stem cell-derived cells were exposed to 1800 MHz RF-EMF at a SAR value of 4 W/kg for 48 h. Each condition contained three parallel samples from independent cultures. Library preparation and sequencing were carried out by the Majorbio Institute (Shanghai, China). Briefly, total RNA was extracted with TRIzol^®^ reagent (Invitrogen, United States). Library construction was done with the Truseq^TM^ RNA sample prep Kit (Illumina, United States). The mRNA sequencing (RNA-seq) was done on the HiSeq 4000 platform (Illumina). The software RSEM and EdgeR were used to quantify the expression levels and differentially expression (DE) of transcripts (Li and Dewey, 2011). The following cut-off criteria were used to filtered the DE transcripts: divergence probability ≥ 0.8, *P*adjust < 0.1, fold change ≥1.5 or ≤−1.5. Followed, Gene Ontology (GO) enrichment and Kyoto Encyclopedia of Genes and Genomes (KEGG) pathway analyses based on these DE transcripts were performed using DAVID^1^ and Blast2GO^2^.

### Neurite Outgrowth Analysis

The neurite outgrowth of cells was monitored and measured in the Incucyte live-cell analysis system (Essen BioScience, United States). Briefly, the RF-EMF exposed cells were dissociated and cultured in 96-well plates. For better identification, the density of NSC-derived cells was adjusted to 15000 cells/well, and Neuro-2A cells were cultured at a density of 4000 cells/well. The growth of cells was monitored for 48 h. The length and branch point of the neurite were quantified.

For neurite staining and morphology observation, cells were cultured in 24-well plates on round coverslips coated with poly-L-lysine. The density of NSC-derived cells was adjusted to 1 × 10^5^ cells/well, and the density of Neuro-2A cells was adjusted to 4 × 10^4^ cells/well.

### Western Blot

Cells were collected at indicated time points and lysed with RIPA buffer (Thermo, United States). Protease inhibitors (Roche, United States) and phosphorylase inhibitors (Roche, United States) were added to the buffer before use. Western blot was quantified with an Odyssey infrared imaging system (LI-COR, United States). The following antibodies were used: mouse anti-ACTB (1:5000, Sigma-Aldrich), mouse anti-DCX (1:1000, Santa Cruz, United States), rabbit anti-EPHA5 (1:500, Invitrogen), mouse anti-CREB (1:1000, Pierce, United States), rabbit anti-p-CREB (1:1000, CST, United States), and rabbit anti-GAPDH (1:1000, GeneTex, United States). The Odyssey-specific second antibodies were IRDye^®^ 680 or 800 donkey anti-rabbit antibody, IRDye^®^ 680 or 800 donkey anti-mouse IgG antibody (LI-COR, United States).

### RhoA GTPase Activation Assay

The level of activated RhoA GTPases in cells after RF-EMF exposure was determined with an active Rho detection kit purchased from Cell Signaling Technology according to previous work (Sin et al., 2020). Cells were collected and lysed in 1× lysis buffer containing 1 mM PMSF. Followed this, the lysates were harvested by centrifuge at 16,000 × g at 4°C for 15 min. The supernatants were incubated with glutathione-S-transferase (GST) agarose beads coupled to Rhothekin RBD recombinant protein, which is used to bind the activated form of GTP-bound RhoA. Then, the GTP-bound RhoA was immunoprecipitated with glutathione resin. Activated RhoA GTPases pull-downs were released by boiling for 5 min in a 2× SDS Sample Buffer with 200 mM dithiothreitol. Bound RhoA was detected by western blot with rabbit anti-RhoA (1:1000, CST, United States).

### Real-Time PCR

Real-time PCR was performed and quantified as we previously described (Livak and Schmittgen, 2001; Chen et al., 2017). The following primers were used: *Epha4* fwd tcgtttctctttggaatttgcg and rev ataatgctcacttcctcccac, *Epha5* fwd ggacgtgccttctcttgtg and rev cttcaccaatctcttcccacc, *Epha7* fwd agaaggagagtggctagtacc and rev ggacaacgagaacactggag, *Epha8* fwd gcgaagtgaacttgttggatac and rev tgcatacttggtacgtgtgg, *Ephb1* fwd cgatggaagagacattgatggac and rev ggtaagtacggatggtgttcag, *Ephb3* fwd actctcatggacacgaaatgg and rev tcgactcacgcacattacac, and *Ephb4* fwd gatcgcattcagccaaagtg and rev aagtcacccatttcagatccg.

### Immunostaining

For immunostaining, cells were cultured on poly-L-lysine-coated round coverslips. Then the cells were fixed with 4% paraformaldehyde at indicated time points. Immunostaining was carried out with the following primary antibodies: rabbit anti-TUBB3 (1:100, GeneTex), mouse anti-GFAP (1:100, Abcam, United States), rabbit anti-ALDH1L1 (1:100, CST), rabbit anti-EPHA5 (1:100, Invitrogen), rabbit anti-SOX2 (1:200, Abcam), mouse anti-NESTIN (1:200, Millipore, United States), and mouse anti-TUBB3 (1:100, R&D, United States). The secondary antibodies used were Alexa Fluor 488-, 555-, and 647-labeled goat anti-mouse and goat anti-rabbit secondary antibodies (1:200, Invitrogen). Cell nuclei were visualized by 5 μg/ml Hoechst33342 (Sigma-Aldrich) staining.

For EdU staining, cells were exposed to EdU (20 mM) for 24 h. EdU staining was carried out with a Cell-Light EdU DNA cell proliferation kit (RiboBio, Guangzhou, China) according to the instructions.

### Phosphoprotein Profile of Key Signaling Pathway by an Antibody Array

Neural stem cells were differentiated under 4 W/kg RF-EMF exposure for 48 h. The changes of signaling pathways were detected by a Phospho Explorer Antibody Array (Full Moon Biosystems, CSP100^*plus*^, United States). Data collection and analysis were carried out by Wayen Biotechnologies (Shanghai, China). Briefly, 452 proteins in 16 signaling pathways were analyzed. The analyzed results were first normalized by housekeeping protein as phosphorylation-protein/housekeeping and total protein-expression/housekeeping. The ratio of protein expression and phosphorylation change after RF-EMF exposure was obtained by comparing it to the control.

### *Epha5* siRNAs Transfection, EPHA5 Recombinant Protein or Forskolin Treatment

All siRNAs were transfected with Lipofectamine^TM^ RNAiMAX transfection reagent (Invitrogen) following the instructions. *Epha5* specific siRNAs and control siRNAs were obtained from Santa Cruz (sc-39939, sc-37007, and sc-36869). The siRNAs were transfected after NSCs differentiated for 2 days. After 2 days of transfection, the neurite outgrowth of NSC-differentiated cells was monitored by the Incucyte live-cell analysis system for another 48 h.

Eph receptors 5 recombinant protein or Forskolin (FSK) treatment was carried out 2 h before RF-EMF exposure. EPHA5 recombinant protein (Thermo Fisher Scientific) was added to the medium at a final concentration of 20 ng/ml. FSK (Sigma-Aldrich) was used at a final concentration of 10 μM.

### Statistical Analyses

All data were routinely collected from three independent duplicate experiments and presented as means ± standard error of the mean (S.E.M.). The repeat number of experiments was increased according to sample variations. Data analysis was conducted with GraphPad Prism 8 software. One-way ANOVA or two-way repeated measures ANOVA with Bonferroni’s *post hoc* test was used for 3 or more independent variables. A two-tailed Student’s *t*-test was used for comparing two sets of data. A *p*-value less than 0.05 was considered statistically significant.

## Results

### Characteristics of the Cultured NSCs

**FIGURE 1:**
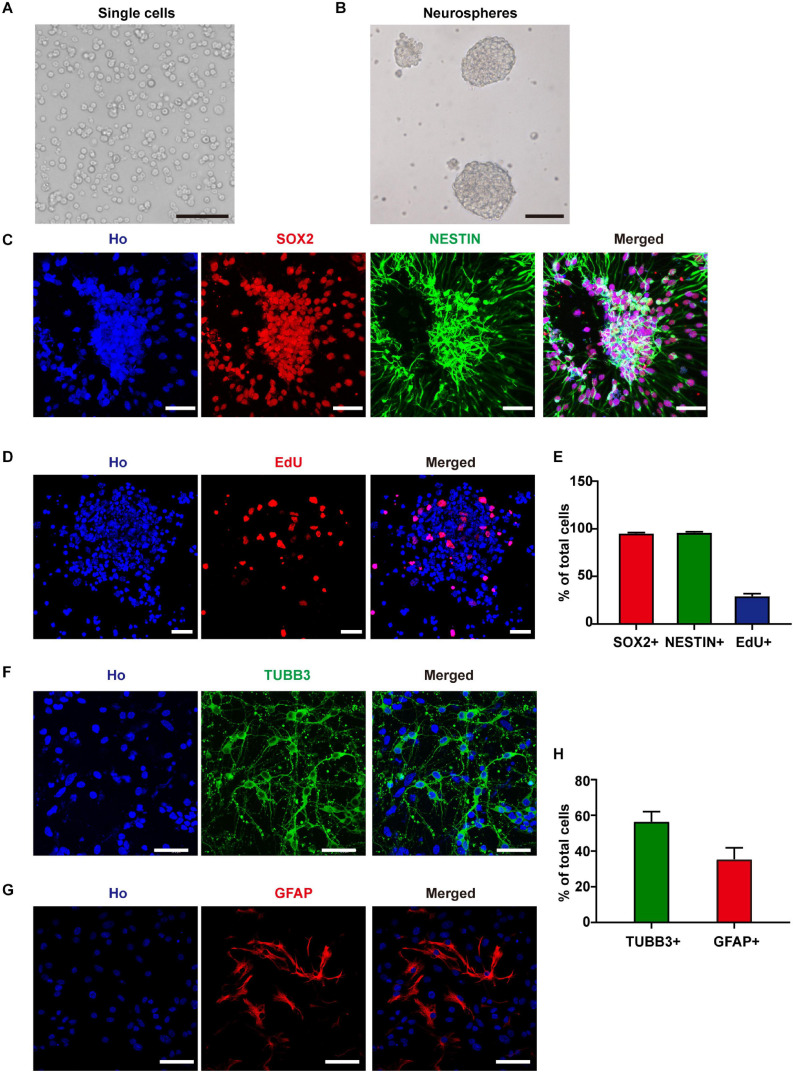
Neural stem cells (NSCs) identification and differentiation. **(A)** Cells isolated from E11.5 mouse telencephalons. Scale bar, 100 μm. **(B)** The single cells formed neurospheres after 6 days of *in vitro* culture. Scale bar, 100 μm. **(C)** SOX2 and NESTIN staining of cultured neurospheres. Scale bar, 50 μm. **(D)** EdU staining of cultured neurospheres. Scale bar, 50 μm. **(E)** Statistic data of the percentage of SOX2^+^, NESTIN^+^, and EdU^+^ cells. **(F–H)** TUBB3 and GFAP staining and statistic data of NSC-derived cells. Scale bar, 50 μm.

To investigate the effects of RF-EMF exposure on neuron development, we established an NSC-based cell model. After 6 days of *in vitro* culture, the isolated single cells formed neurospheres (Figures 1A,B). Besides, more than 95% of the cells in neurospheres expressed NSC markers SOX2 and NESTIN (Figures 1C,E). After EdU incorporation for 24 h, about 29.1% of these cells were EdU^+^, which indicated a high ratio of DNA synthesis and cell proliferation (Figures 1D,E). Next, we induced the cells differentiated in the normal condition, in which the medium contained 1% FBS and 1 μM RA without bFGF and EGF. We could get 56.5% of TUBB3^+^ neurons and 35.4% of GFAP^+^ astrocytes after 4 days of *in vitro* differentiation (Figures 1F–H). The morphology observation of neurons and astrocytes in the living condition suggested that neurites of neurons were strong and identifiable after 4 days of *in vitro* differentiation (Supplementary Figure 1). The soma of astrocytes was big and flat relative to neurons and they grew in the bottom layer of the well (Supplementary Figure 1). The process of astrocytes is unidentifiable due to their growth characteristics (Supplementary Figure 1).

### RF-EMF Exposure Induced Transcriptomic Changes Related to Neurite Outgrowth

**FIGURE 2:**
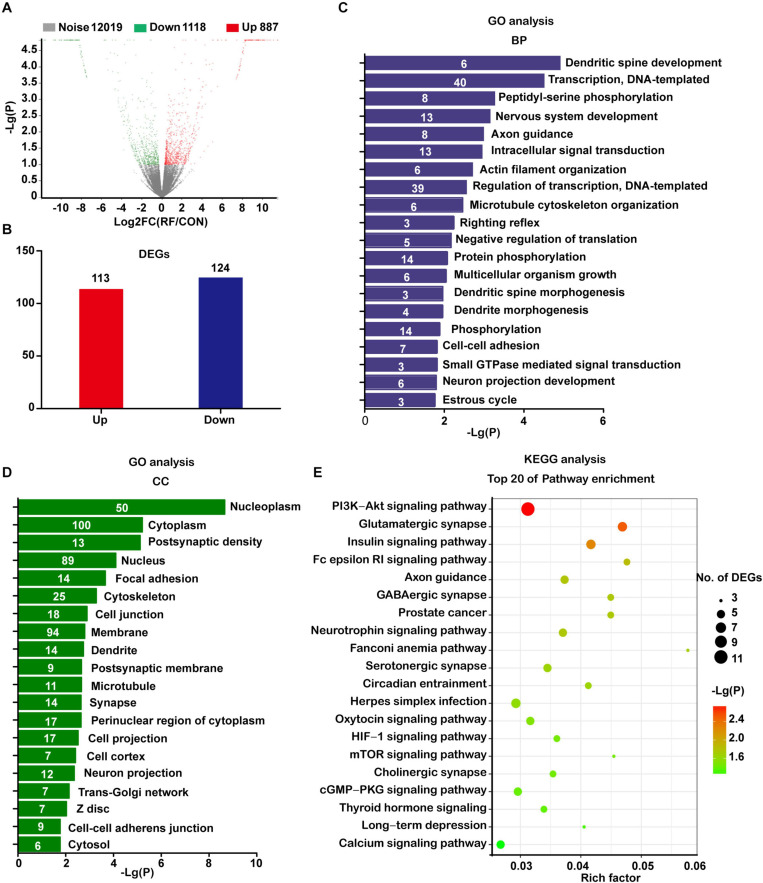
Transcriptomic profile of NSC-differentiated cells after 1800 MHz RF-EMF exposed for 48 h. **(A)** The volcano graph shows all the DE transcripts with the criteria condition of *P*adjust < 0.1 and divergence probability ≥ 0.8. **(B)** DE transcripts filtered by the criteria: *P*adjust < 0.1, divergence probability ≥ 0.8 and |FC| ≥ 1.5. **(C,D)** Results of top items of biological process (BP) and cellular component (CC) from GO analyses. The number in the bar represents the number of DE transcripts in each item. **(E)** Bubble chart showing the results of the top 20 KEGG pathways. Rich factor refers to the ratio of DE transcripts in KEGG pathways.

Next, we used RNA-seq to analyze the global transcriptomic changes induced by RF-EMF exposure. The NSC-derived cells were exposed to 1800 MHz 4 W/kg RF-EMF for 48 h. Differentially expressed (DE) transcripts between the RF-EMF exposure group and the control group were filtered using NOISeq. The volcano graph shows all the DE transcripts with the criteria condition of *P*adjust < 0.1 (Figure 2A). When adding another filter criteria |FC| ≥ 1.5, the data revealed that only 113 transcripts were up-regulated and 124 transcripts were down-regulated (Figure 2B).

The significantly changed DE transcripts were then chosen for GO and KEGG pathway analysis. The results from the category biological process (BP) revealed that these DE transcripts enriched in dendritic spine development, nervous system development, axon guidance, actin filament organization, microtubule cytoskeleton organization, dendrite morphogenesis, and neuron projection development (Figure 2C). All those items were closely or directly related to axon or dendrite development. Results from the category cellular component (CC) suggested that the most enriched items included post-synaptic density, cytoskeleton, cell junction, dendrite, post-synaptic membrane, microtubule, synapse, cell projection, and neuron projection (Figure 2D).

The KEGG pathway analysis showed that the enriched pathways including axon guidance, the glutamatergic synapse, GABAergic synapse, serotonergic synapse, and neurotrophin signaling pathway. These pathways were directly related to neurite development. Also, the enriched items included some pathways which have been demonstrated closely related to neurite development, such as the PI3K-AKT signaling pathway (Zhao et al., 2020), mTOR signaling pathway (Wen et al., 2020), cGMP-PKG signaling pathway (Sun et al., 2020), thyroid hormone signaling pathway (Poddar et al., 1996), and calcium signaling pathway (Zhao et al., 2019; Figure 2E).

When using a much more strict criteria condition of *P*adjust < 0.05 and |FC| ≥ 2, only 85 up-regulated transcripts and 100 down-regulated transcripts were got. However, the GO and KEGG pathway analysis still revealed similar results with previous analyses (Supplementary Figure 2). Taken together, these data strongly indicated that RF-EMF exposure influenced neurite development.

### RF-EMF Exposure Impairs Neurite Outgrowth

**FIGURE 3:**
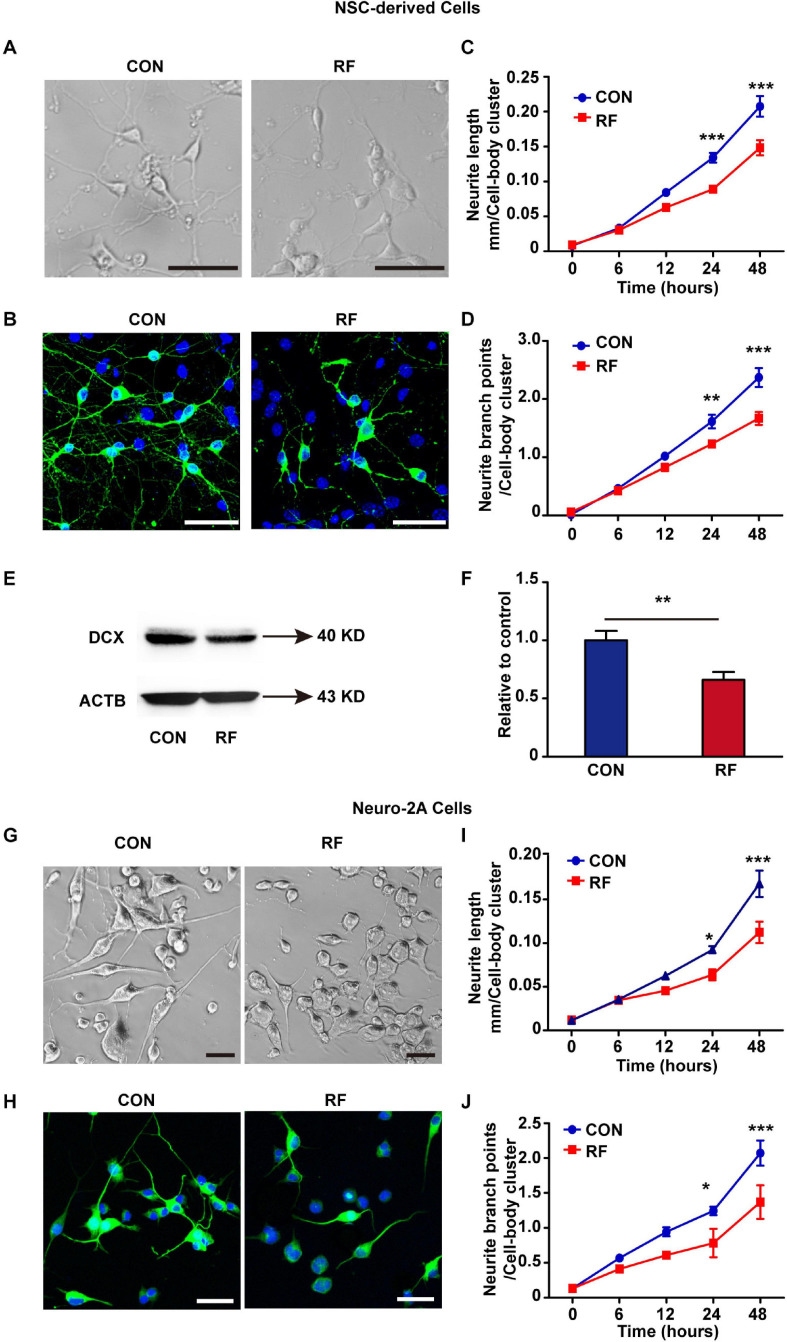
1800 MHz RF-EMF exposure inhibited neurite outgrowth. **(A,B)** NSCs were induced differentiation for 48 h under 4 W/kg RF-EMF exposure. **(A)** Representative phase-contrast images of NSC-differentiated cells after RF-EMF exposure. Scale bar, 50 μm. **(B)** TUBB3 staining of NSC-differentiated neurons. Scale bar, 50 μm. **(C,D)** The neurite outgrowth of NSC-differentiated cells was monitored by the Incucyte live-cell analysis system. **(C)** The length of the neurite was reduced remarkably after RF-EMF exposure. **(D)** Neurite branch points were decreased after RF-EMF exposure. **p < 0.01, and ***p < 0.001, two-way repeated measures ANOVA followed by Bonferroni post-tests. **(E,F)** RF-EMF exposure decreased the protein expression of DCX in NSC-derived cells. **p < 0.01 by Student *t*-test. **(G,H)** Neuro-2A cells were induced differentiation for 48 h under 4 W/kg RF-EMF exposure. Representative phase-contrast images and TUBB3 staining of the cells. Scale bar, 50 μm. **(I,J)** Neurite length and neurite branch points were decreased after RF-EMF exposure as monitored by the Incucyte live-cell analysis system. *p < 0.05, and ***p < 0.001, two-way repeated measures ANOVA followed by Bonferroni post-tests.

To verify the insights from RNA-seq analysis and further explore how RF-EMF exposure influenced neurite outgrowth, we firstly used embryonic NSCs as a cell model and detected the influence of RF-EMF exposure on the neurite outgrowth in NSC-differentiated neurons. NSC-derived cells were exposed to 1800 MHz 4 W/kg RF-EMF. The phase-contrast images and TUBB3 staining revealed that neurite outgrowth was inhibited remarkably after RF-EMF exposure (Figures 3A,B and Supplementary Figure 3). Besides, the neurite outgrowth was further monitored and quantified by the Incucyte live-cell analysis system for 48 h after exposure. We found that the length of neurite in RF-EMF exposed cells was significantly shorter than control cells from 24 h post-exposure (Figure 3C). In addition, the branch points in RF-EMF exposed cells were reduced compared to control as detected 24 and 48 h post-exposure (Figure 3D). We then detected the protein expression of doublecortin (DCX), as DCX is a microtubule-associated protein required for the initial steps of neurite outgrowth (Jean et al., 2012; Fu et al., 2013). The results showed that DCX expression was down-regulated remarkably after RF-EMF exposure (Figure 3E,F).

To further confirm the results, we used another cell model Neuro-2A cell. 10 μM RA was used to treat the cells to induce the cells to differentiate into a neuronal-like morphology. The morphology showed that neurite outgrowth was significantly inhibited in RF-EMF exposed cells (Figures 3G,H). We also found notable reductions in both the length of neurite and the number of branch points in RF-EMF exposed cells (Figures 3I,J). Together, these data revealed that 1800 MHz RF-EMF exposure has significant inhibitory effects on neurite outgrowth.

### RF-EMF Exposure Down-Regulates the Expression of EPHA5

**FIGURE 4:**
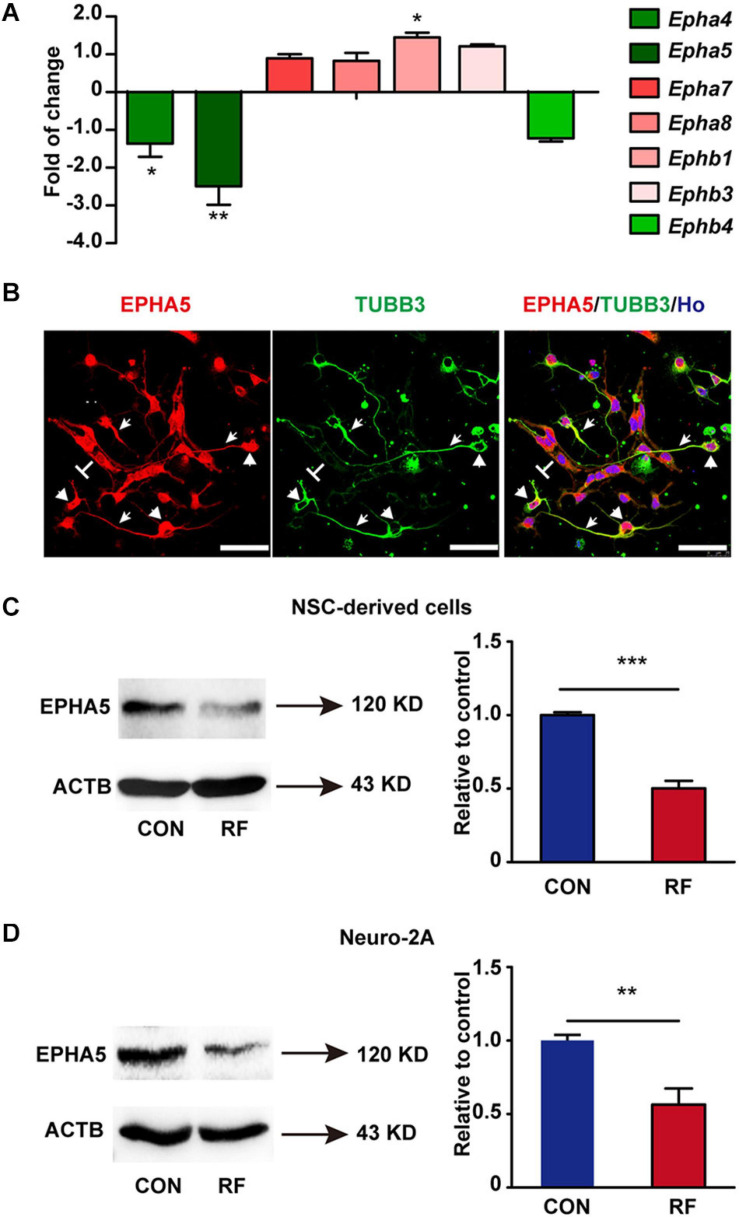
The expression of EPHA5 is reduced after RF-EMF exposure. **(A)** The change of the mRNA expression of Eph receptors after 4 W/kg RF-EMF exposure. The mRNA expression of *Epha5* was significantly decreased after RF-EMF exposure. *p < 0.05, and **p < 0.01 by Student *t*-test. **(B)** EPHA5 is expressed in NSC-derived neurons. Arrowhead showed the expression of EPHA5 in the process of neurons. Triangle showed the expression of EPHA5 in the soma of neurons. “T” showed the non-neuronal like EPHA5^+^ cells. Scale bar, 50 μm. **(C,D)** The protein expression of EPHA5 is down-regulated in NSC-derived cells and Neuro-2A cells after RF-EMF exposure. **p < 0.01, and ***p < 0.001 by Student *t*-test.

From the results of RNA-seq, we found that Eph receptors (Eph), which regulate fundamental developmental processes of neurite outgrowth (Fiederling et al., 2017; Huang et al., 2018; Fiore et al., 2019), were significantly influenced after RF-EMF exposure. Thus, we then verified the alteration of the mRNA expression of Eph receptors. The mRNA expressions of *Epha4*, *Epha5*, *Epha7*, *Epha8*, *Ephb1*, *Ephb3*, and *Ephb4* were detected because these Eph receptors have been found expressed in the brain previously (Goldshmit et al., 2006; Hruska and Dalva, 2012). Besides, some of those Eph receptors have also been detected in our RNA-seq analysis. Among these factors, the expression of *Epha5* was the most significantly inhibited after RF-EMF exposure (Figure 4A). *Epha4* and *Ephb1* were slightly influenced. The mRNA expression changes of other Eph receptors did not reach a statistical significance. Next, the protein expression of EPHA5 in NSC-differentiated cells was detected. We found that EPHA5 was highly expressed in the soma and process of TUBB3^+^ neurons (Figure 4B). EPHA5 was also found expressed in some cells which did not have neuronal morphology (Figure 4B). Besides, the protein expression of EPHA5 was notably decreased in NSC-differentiated cells after RF-EMF exposure (Figure 4C). This result was further confirmed in RA-induced Neuro-2A cells, in which a remarkable reduction of EPHA5 protein expression was found after RF-EMF exposure (Figure 4D).

### RF-EMF Inhibits Neurite Outgrowth by Down-Regulating EPHA5 Expression

**FIGURE 5:**
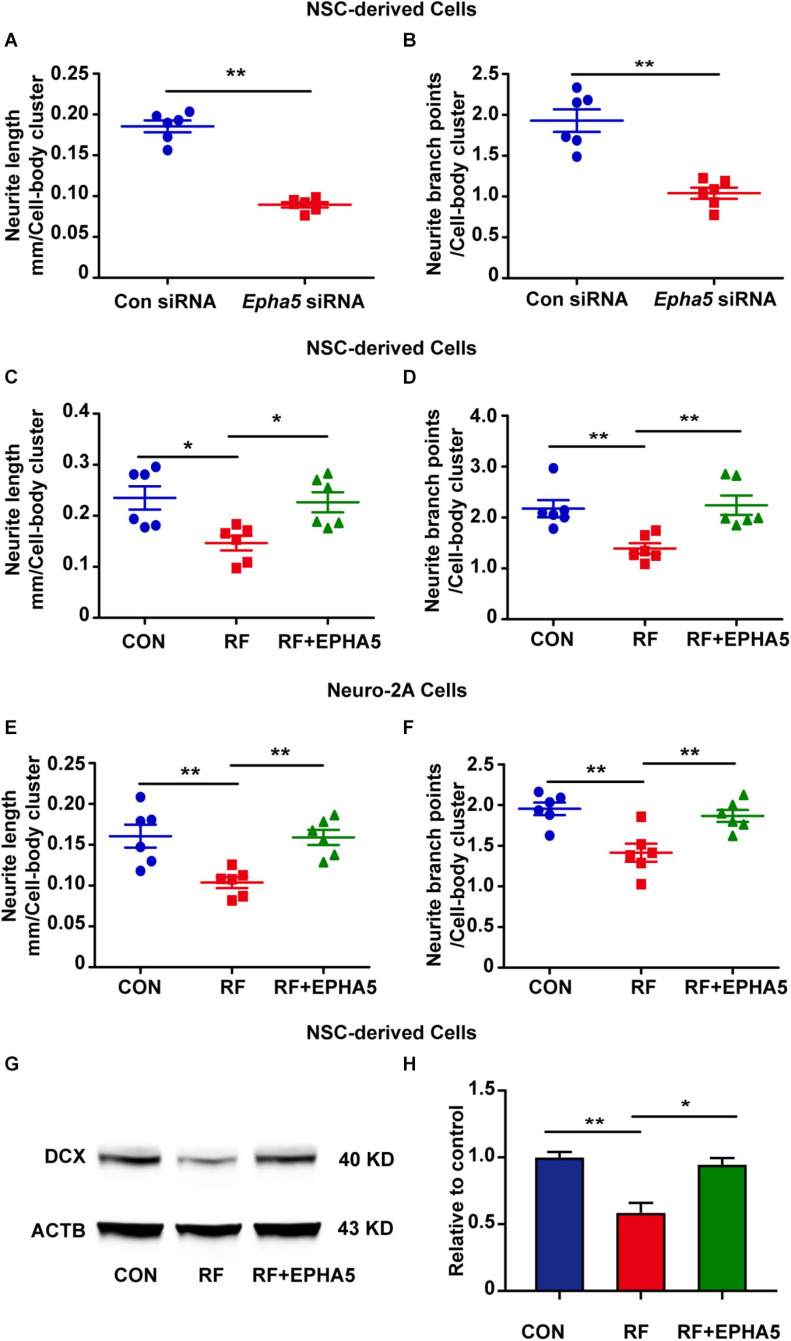
Eph receptors 5 (EPHA5) plays a critical role in mediating the effects of RF-EMF on neurite outgrowth. **(A,B)** EPHA5 silencing decreased neurite length and branch points of NSC-derived neurons. **p < 0.01 by Student *t*-test. **(C,D)** EPHA5 recombinant protein treatment rescued the inhibitory effects of RF-EMF on neurite outgrowth in NSC-derived neurons. *p < 0.05, and **p < 0.01, One-way ANOVA followed by Bonferroni post-tests. **(E,F)** EPHA5 recombinant protein treatment rescued neurite outgrowth in Neuro-2A cells. **p < 0.01, one-way ANOVA followed by Bonferroni post-tests. **(G,H)** EPHA5 recombinant protein treatment rescued the protein expression of DCX after RF-EMF exposure. *p < 0.05, and **p < 0.01, One-way ANOVA followed by Bonferroni post-tests.

It is reported that EPHA5 plays a key role in the developing brain (Teng et al., 2017; Suo et al., 2018). To confirm the role of EPHA5 on neurite outgrowth, we used specific siRNAs to silence EPHA5 expression in NSC-differentiated cells (Supplementary Figure 4). We found that EPHA5 silencing resulted in a notable decrease in the length and branch points of neurites (Figures 5A,B). These results confirmed that EPHA5 is required for neurite outgrowth during neuron development. We then used an EPHA5 recombinant protein to treat the cells during RF-EMF exposure to antagonize the inhibitory effects of RF-EMF on neurite outgrowth. Strikingly, EPHA5 recombinant protein treatment remarkably reversed the neurite outgrowth in both RF-EMF exposed NSC-derived neurons and RA-induced Neuro-2A cells (Figures 5C–F). Besides, EPHA5 recombinant protein treatment also rescued DCX protein expression in RF-EMF exposed NSC-derived neurons (Figures 5G,H). Together, these results revealed that EPHA5 played a key role in mediating the effects of RF-EMF on neurite outgrowth.

### CREB and RhoA Are Involved in EPHA5 Signaling Which Mediates the Effects of RF-EMF

**FIGURE 6:**
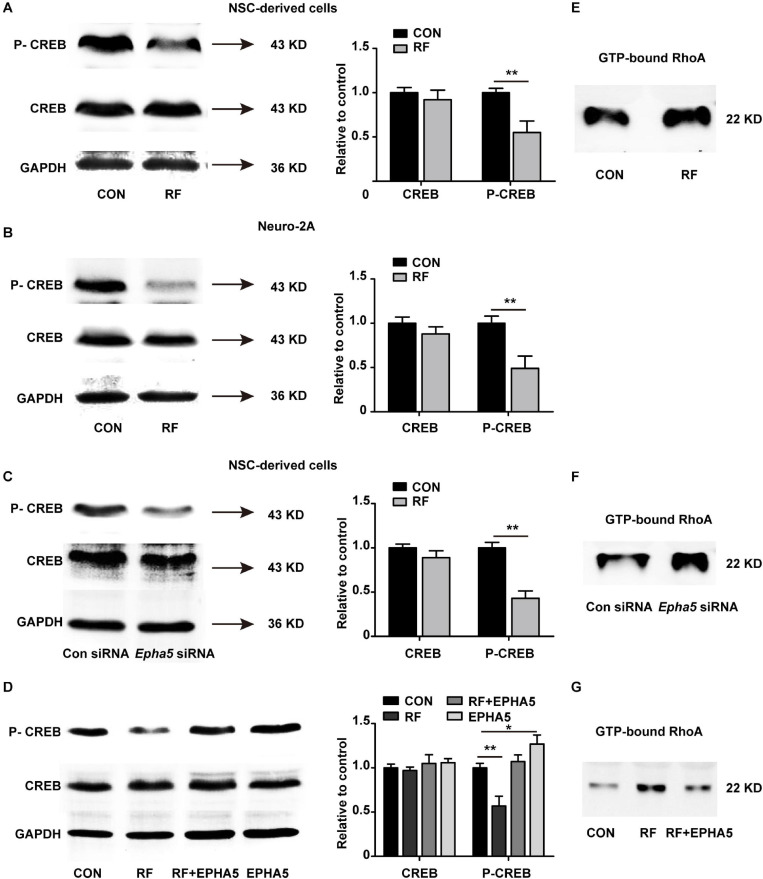
Radiofrequency electromagnetic fields (RF-EMF) exposure decreased CREB phosphorylation through EPHA5. **(A)** RF-EMF exposure inhibited CREB phosphorylation in NSC-derived cells. **p < 0.01 by Student *t*-test. **(B)** RF-EMF exposure reduced the phosphorylation of CREB in Neuro-2A cells. **p < 0.01 by Student *t*-test. **(C)** EPHA5 silencing decreased CREB phosphorylation in NSC-derived cells. **p < 0.01 by Student *t*-test. **(D)** EPHA5 recombinant protein treatment rescued CREB phosphorylation after RF-EMF exposure in NSC-derived cells. *p < 0.05, and **p < 0.01 by Student *t*-test. **(E)** RF-EMF exposure increased the GTP-bound RhoA level. **(F)** EPHA5 silencing increased the GTP-bound RhoA level. **(G)** EPHA5 recombinant protein treatment reversed the GTP-bound RhoA level after RF-EMF exposure.

To further detect the downstream factors through which EPHA5 regulated neurite outgrowth, we carried out a phospho-specific protein microarray to explore the protein expression and phosphorylation change of key factors from 16 signaling pathways. Briefly, 1318 antibodies against phosphorylation sites of 452 proteins were detected. The Core Signal-net was analyzed based on protein expression and phosphorylation change after RF-EMF exposure. The results suggested that CREB signaling might play a key role in RF-EMF-induced inhibitory effects on neurite outgrowth (Supplementary Figure 5). We then detected the effects of RF-EMF exposure on the phosphorylation of CREB by western blot in differentiated NSCs and Neuro-2A cells. The phosphorylation of CREB was inhibited robustly both in NSCs (Figure 6A) and Neuro-2A cells (Figure 6B), while the total protein expression of CREB was not significantly changed (Figures 6A,B). These results revealed that RF-EMF exposure inhibited CREB phosphorylation. Next, we detected CREB phosphorylation in EPHA5 silencing and EPHA5 recombinant protein-treated NSC-derived cells. The results suggested that EPHA5 silencing led to a remarkable reduction of CREB phosphorylation (Figure 6C). Besides, EPHA5 recombinant protein treatment enhanced CREB phosphorylation and antagonized the inhibitory effects of RF-EMF on CREB phosphorylation (Figure 6D).

The RhoA pathway is previously demonstrated associating with dendrite development and axonal extension (Tan et al., 2020). Also, our RNA-seq analysis indicated that the Rho GTPase binding activity could be influenced after RF-EMF exposure. We then analyzed RhoA activation by detecting the GTP-bound RhoA in RF-EMF exposed cells. We found that RF-EMF exposure increased the level of GTP-bound RhoA (Figure 6E). Besides, EPHA5 silencing caused a significant increase in RhoA activation (Figure 6F). Furthermore, the RF-EMF-induced activation of RhoA is inhibited by EPHA5 recombinant protein treatment (Figure 6G). Together, these data indicated that RF-EMF exposure down-regulated CREB phosphorylation and up-regulated RhoA activation through EPHA5.

**FIGURE 7:**
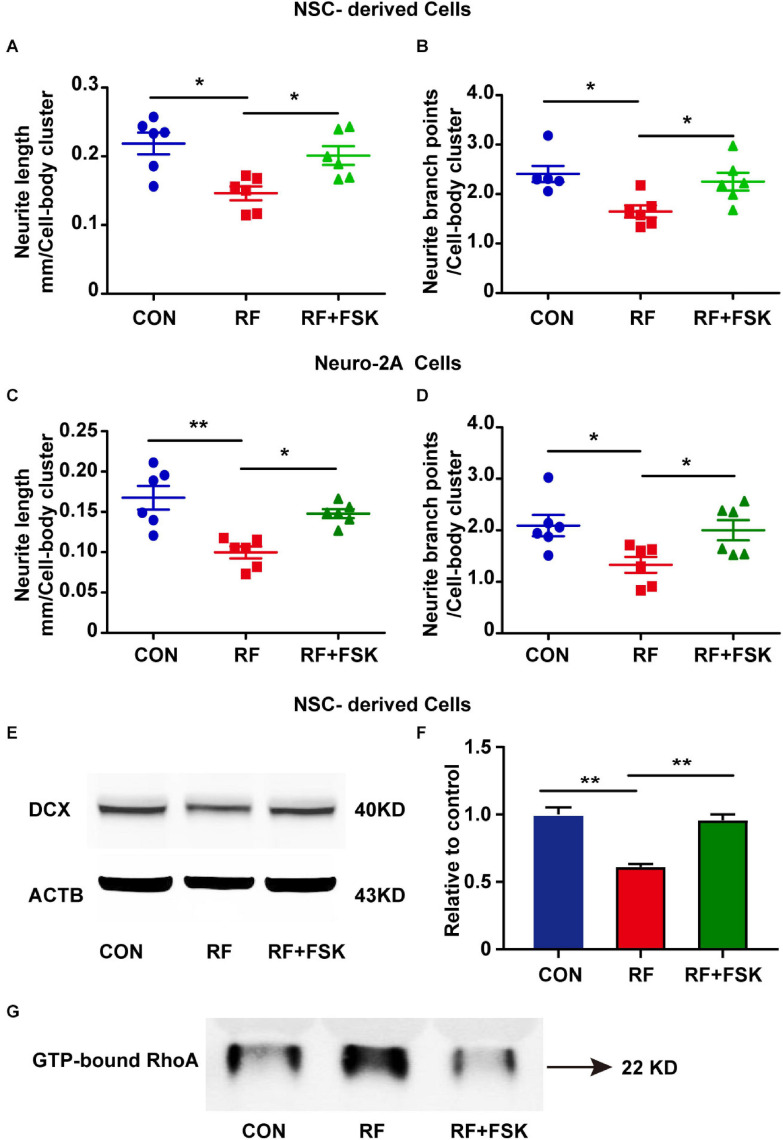
Forskolin (FSK) treatment rescued neurite outgrowth in RF-EMF exposed cells. **(A,B)** FSK treatment antagonized the inhibitory effects of RF-EMF exposure on neurite outgrowth in NSC-derived cells. **(C,D)** FSK treatment rescued neurite outgrowth in Neuro-2A cells after RF-EMF exposure. *p < 0.05, and **p < 0.01, one-way ANOVA followed by Bonferroni post-tests. **(E,F)** FSK treatment rescued the protein expression of DCX. **p < 0.01, One-way ANOVA followed by Bonferroni post-tests. **(G)** FSK treatment reversed the GTP-bound RhoA level after RF-EMF exposure.

Next, we treated the cells with FSK, which is a cAMP activator. Previous investigations have revealed that cAMP activation enhances the phosphorylate of RhoA on Ser188 and inhibits the activation of RhoA (Ellerbroek et al., 2003). FSK is also an activator of CREB (Heinick et al., 2015; Kim et al., 2021). Our data suggested that 10 μM FSK treatment reversed the inhibitory effects of RF-EMF on CREB phosphorylation (Supplementary Figure 6). We found that the reductions in the length and branch points of neurite in RF-EMF exposed cells were reversed by FSK treatment in both NSC-derived cells (Figures 7A,B) and Neuro-2A cells (Figures 7C,D). The reduction of the expression of DCX induced by RF-EMF exposure in NSC-derived cells was also recovered after FSK treatment (Figures 7E,F). Besides, the RF-EMF-induced activation of RhoA was also inhibited by FSK treatment (Figure 7G). The data demonstrated that FSK treatment rescued the neurite outgrowth in RF-EMF exposed cells. It further demonstrated that CREB and RhoA were downstream factors of EPHA5 through which RF-EMF inhibited neurite outgrowth.

## Discussion

The influences of RF-EMF exposure on brain development have attracted great public concern. The biological effects of RF-EMF exposure on brain development have not yet been well addressed. Besides, the underlying mechanisms have not been well explored. In this study, we revealed the inhibitory effects and its underlying mechanisms of 1800 MHz RF-EMF exposure on the development of neurite based on a high-throughput RNA-seq analysis.

It is important to uncover the hazard effects and the underlying mechanisms of RF-EMF on the developing brain, considering the greater susceptibility of the developing brain, the greater penetration of RF-EMF and thin skull’s bone, and their potential for a longer lifetime exposure (Redmayne et al., 2013; Fernandez et al., 2018). Previous studies have revealed that the developing brain is one of the major targets influenced by RF-EMF exposure (Kaplan et al., 2016; Kim et al., 2019a). To better explore the effects of RF-EMF exposure on brain development, we used an embryonic NSC-based cell model. From the results of RNA-seq analysis in the embryonic NSC model, we found that transcripts related to the key processes of brain development, including axon and dendrite development, projection, and synapses formation, were significantly changed. The results confirmed that brain development is highly sensitive to RF-EMF exposure. Particularly, many remarkable altered items detected by GO and KEGG analysis targeted neurite outgrowth, indicating that the development of neurite was a crucial target through which RF-EMF acts on the developing brain.

The hazardous effects of RF-EMF exposure on the development of the brain have been investigated in human and animal models. Studies have addressed that long-term RF-EMF exposure causes cell loss in the developing brain. Postnatal exposure to 2 W/kg 900 MHz RF-EMF for 1 h/day 21 days decreases the pyramidal cell number in the hippocampus and Purkinje cell number in the cerebellum in rats (Bas et al., 2009; Sonmez et al., 2010). Prenatal exposure to 2 W/kg 900 MHz RF-EMF for 1 h/day 21 days reduces the number of granule cells in the dentate gyrus in the rat hippocampus (Odaci et al., 2008). Other researchers found that RF-EMF exposure does not cause cell apoptosis (Joubert et al., 2007; Zhou et al., 2019). These inconsistent results suggested that much more studies are requested to get a robust conclusion, particularly to address much finer changes in cells except for cell death during brain development. Lots of studies have demonstrated that RF-EMF exposure impairs cognitive functions. Thus, it is necessary to explore the influence of RF-EMF on neurite outgrowth. Continuous exposure to 900 MHz 1 W/kg RF-EMF for 48 or 72 h reduces neurite numbers generated by murine SN56 cholinergic cell line and rat primary cortical neurons (Del Vecchio et al., 2009). 1800 MHz 4.0 W/kg RF-EMF exposure for 24 h reduces the length of the axon branch and the number of branches in cortical neurons (Su et al., 2018). Here, we used mouse embryonic NSCs to generate newborn neurons. The cell model is much more close to the developing brain *in vivo*. The data from this *in vitro* cell model demonstrated that neurite outgrowth is inhibited after 1800 MHz RF-EMF exposure. Furthermore, this conclusion was confirmed in another cell model Neuro-2A cell. Together, these data suggested that neurite outgrowth is a key process influenced by RF-EMF during brain development. The results also emphasized that much more investigations focus on the influence of RF-EMF on neurite development are needed to fully explore the effects and mechanisms. Also, based on our RNA-seq results and previous report (Kim et al., 2019a), some other key processes including neurite outgrowth such as synapses and dendritic spine development need further studies to address.

Exploring the mechanisms of how RF-EMF affects neurite outgrowth has important implications to fully understand how RF-EMF acts on brain development. Till now, more evidence is still needed to fully understand how cells physically sense RF-EMF. Previous studies have explored that RF-EMF exposure inhibits calcium influx, causes oxidative stress, and induces DNA injury (Altun et al., 2018; Kim et al., 2018a). These effects might cause downstream effects such as changing the expression of specific genes, modifying the morphology of the nervous system, and even leading to impairment of cognitive functions (Keles et al., 2018; Kim et al., 2019a; Narayanan et al., 2019). The Eph receptors are multitalented tyrosine kinases that perform many tasks. It has been explored that Eph receptors play an important role in the initial assembly of neuronal circuits during embryonic brain development (Soskis et al., 2012; Teng et al., 2017). As indicated by our RNA-seq results, Eph receptors were remarkably influenced by RF-EMF. Among these factors, EPHA5 was the most sensitive factor that responded to RF-EMF. Here, we did not address the mechanisms that how EPHA5 was inhibited by RF-EMF. However, it is reported that T-type calcium channel regulates ephrin-A/EPHA expression during neural development (Abdul-Wajid et al., 2015). The research indicates that RF-EMF might regulate EPHA5 through calcium signaling.

During embryonic brain development, EPHA5 protein is found highly expressed in the cerebral neocortex, hippocampus, pretectum, tectum, and olfactory bulb (Cooper et al., 2009). Here, we found that EPHA5 is highly expressed in the neurites of NSC-derived neurons. Besides, down-regulation of EPHA5 signaling remarkably inhibited neurite outgrowth in NSC-derived neurons, confirming the role of EPHA5 in regulating the outgrowth of neurite in newborn neurons. Since RF-EMF exposure significantly decreased the protein expression of EPHA5, we used EPHA5 recombinant protein to enhance EPHA5 signaling. The results suggested that EPHA5 activation significantly rescued neurite outgrowth after RF-EMF exposure. These data strongly demonstrated that RF-EMF inhibited neurite outgrowth through EPHA5 signaling.

Eph receptors 5 has been revealed to play critical roles in regulating neuronal spine structure, growth cone repulsion, and synaptogenesis during brain development (Brennaman et al., 2014; Das et al., 2016). EPHA5 signaling is also found involved in the region-specific targeting of raphe serotonin neurons (Teng et al., 2017). Besides, the formation of the ascending midbrain dopaminergic pathways is also regulated by EPHA5 (Kimura et al., 2011). It has also been revealed that abnormal expression of EPHA5 affects synaptogenesis in congenital hypothyroidism rats during brain development (Suo et al., 2018). In our experiments, we also found that EPHA5 was expressed in cells that did not have a neuronal morphology. These cells were probably astrocytes according to their morphology. The finding indicated that EPHA5 in astrocytes might play a role in neurite outgrowth through neuron-astrocyte interaction. However, further investigations were needed to confirm this possibility. Together, these findings indicated that RF-EMF might affect multiple processes of brain development which deserve further study.

Previous investigations have identified that the Eph receptors as cell surface receptors that are required for developing neurons to detect environmental cues and respond to them (Beg et al., 2007). However, the downstream signaling mechanisms of the Eph receptors are only poorly understood. Here, we used an antibody array to screen out the potential downstream signaling of EPHA5 and found that CREB was a critical downstream factor of EPHA5. The previous study revealed that activation of EphB2 enhances CREB activation in mice brain (Alapin et al., 2018). The ephrinB1-EphB signaling is revealed to activate CREB through protein kinase A in the spinal cord (Zhou et al., 2015). Besides, ephrin-A5 interaction with its receptor EPHA5 activates CREB, which plays a key role in synaptogenesis during neuronal development (Akaneya et al., 2010).

Besides, we found that RhoA played a key role together with CREB through which EPHA5 regulated neurite outgrowth after RF-EMF exposure. RhoA is a critical molecular which inhibits neurite outgrowth by regulating the actin cytoskeleton (Fujita and Yamashita, 2014). It was found that inhibition of RhoA blocked the effects of EphB2-induced axonal retraction in hippocampal neurons (Takeuchi et al., 2015). EphA1 modulates cell spreading and migration through RhoA-ROCK signaling, which is important for the regulating of cell morphology (Yamazaki et al., 2009). RhoA is also found required for the ephrinB3/EphA4-dependent effects on assembling cortical and spinal motor circuits (Mulherkar et al., 2013). These studies indicated that CREB and RhoA could be downstream factors of EHPA5 signaling which mediated the effects of RF-EMF. We also proved that FSK treatment antagonized the inhibitory effects of RF-EMF on neurite outgrowth. FSK is a cAMP activator, which can enhance the phosphorylation and activation of CREB (Lv et al., 2019). It was found that cAMP activation enhances the phosphorylate of RhoA on Ser188 and inhibits the activation of RhoA (Ellerbroek et al., 2003). Our results revealed that FSK treatment reversed the effects of RF-EMF on RhoA activation and CREB phosphorylation. Thus, the rescue effects of FSK on neurite outgrowth demonstrated that CREB and RhoA are downstream factors of EPHA5. RF-EMF inhibited neurite outgrowth through EPHA5-CREB/RhoA signaling. Besides, it is still unknown whether FSK treatment or CREB activation could act on EPHA5 expression. To address this object, further research is needed.

The SAR we used here was based on the results of our previous studies (Chen et al., 2014) and the International Commission on Non-Ionizing Radiation Protection (ICNIRP) Guidelines. Based on the ICNIRP Guidelines 2020, the basic restrictions for EMF exposure from 100 KHz to 6 GHz are set at a whole-body average SAR of 0.4 W/Kg for occupational exposure and 0.08 W/Kg for general public exposure for average intervals longer than 6 min (ICNIRP, 2020). Besides, these thresholds have taken a reduction factor of 10 for occupational exposure and 50 for public exposure from SAR value of 4 W/Kg, at which level adverse effects could be observed (ICNIRP, 2020). Thus, our results provide new evidence for better understanding the ICNIRP Guidelines and for the necessity to restrict RF-EMF exposure at a safe level.

## Conclusion

In conclusion, the effects of RF-EMF exposure on brain development have not been well addressed yet. Particularly, the mechanisms of how RF-EMF exposure affects brain development are largely unknown. In this study, we used a previously established NSC-derived neuron development model, and high-throughput RNA-seq methods combined with an antibody microarray to screen the effects and mechanisms of RF-EMF on neuron development. The finding revealed that RF-EMF remarkably impaired neurite outgrowth through EPHA5-CREB/RhoA signaling. This finding contributes to revealing the effects and mechanisms of RF-EMF on neurite outgrowth. Besides, it also shed light on our further studies of exploring the effects and mechanism of RF-EMF on brain development.

## Data Availability Statement

The datasets presented in this study can be found in online repositories. The names of the repository/repositories and accession number(s) can be found below: SRA; PRJNA694499.

## Ethics Statement

The animal study was reviewed and approved by Third Military Medical University.

## Author Contributions

CC, YZ, ZY, and LZ conceived the study. CC, QM, PD, ML, PG, MH, YL, HP, ZH, and CZ performed the experiments. CC, QM, and LZ analyzed the data. CC wrote the manuscript. All authors contributed to the manuscript revision and approved the submitted version.

## Conflict of Interest

The authors declare that the research was conducted in the absence of any commercial or financial relationships that could be construed as a potential conflict of interest.

## Footnotes

1 https://david.ncifcrf.gov/

2 https://www.blast2go.com/
