# Supplementary material for: 1800 MHz Radiofrequency Electromagnetic Field Impairs Neurite Outgrowth Through Inhibiting EPHA5 Signaling
Source: Front Cell Dev Biol. 2021 Apr 12;9:657623. doi: 10.3389/fcell.2021.657623 (PMC8075058; doi:10.3389/fcell.2021.657623)
Supplement: Supplementary file 1 [file Data_Sheet_1.PDF]

SUPPLEMENTARY FIGURE S1

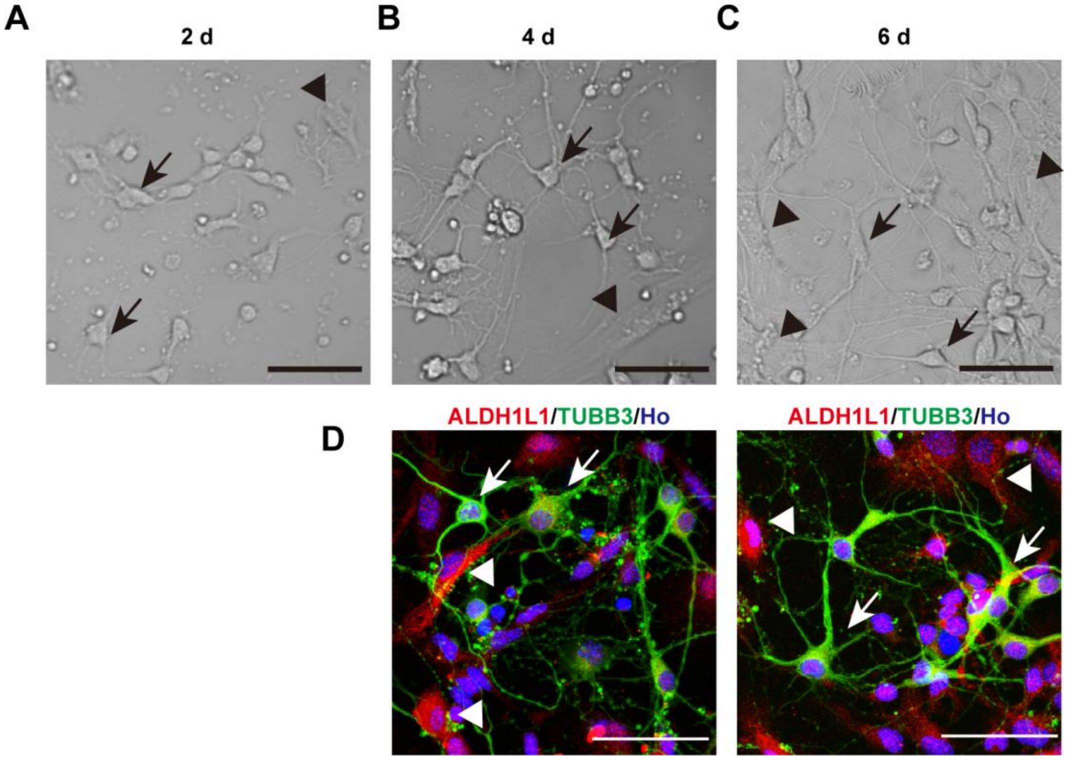

FIGURE S1 Characteristics of NSC-derived cells. (A) Two days of *in vitro* differentiation. The neurites in neurons were short. The morphologies of astrocytes were not typical and the somata were small in size. (B) Four days of *in vitro* differentiation. The neurites in neurons were long. The somata of astrocytes were big and flat relative to neurons. (C) Six days of *in vitro* differentiation. The neurites in neurons were very long. The somata of astrocytes were big and flat. (D) Morphology of differentiated neurons and astrocytes was examined by staining of the neuron-specific marker TUBB3 and astrocyte-specific marker ALDH1L1. Arrowhead showed the differentiated neurons. Triangle showed the differentiated astrocytes. Scale bar, 50 μm.

SUPPLEMENTARY FIGURE S2

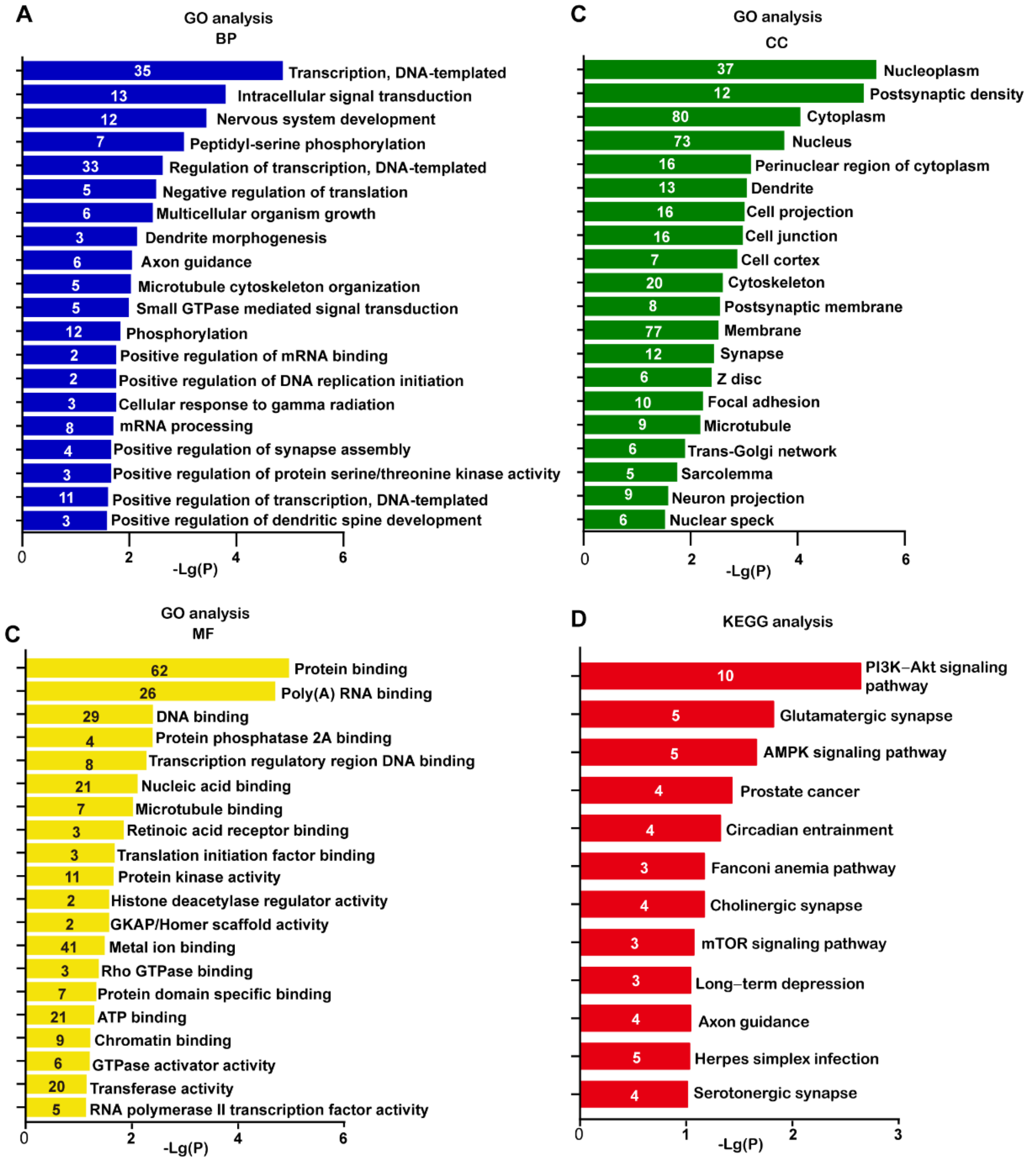

FIGURE S2 GO and KEGG analysis with DE transcripts from criteria condition of Padjust < 0.05 and ∣FC∣≥2. (A) Results of top items of BP (biological process) from GO analyses. (B) Top items of CC (cellular component) from GO analyses. (C) Top items of MF (molecular function) from GO analyses. (D) Top items from KEGG pathway analyses. The number in the bar represents the number of DE transcripts in each item.

SUPPLEMENTARY FIGURE S3

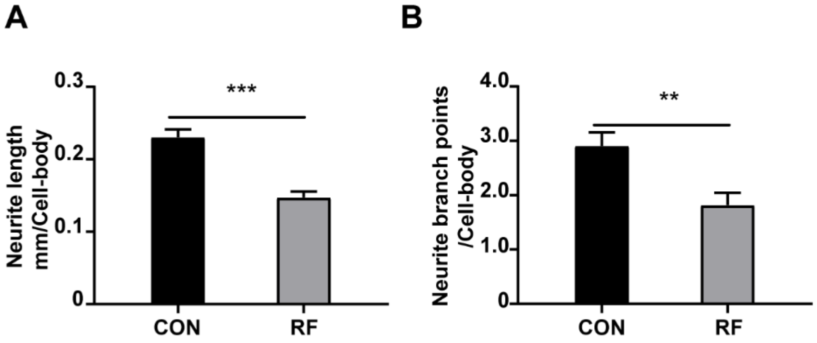

FIGURE S3 Statistic results of TUBB3 staining. (A) The total length of the neurite in control and RF-EMF-exposed cells. (B) The number of neurite branch points in the cells. The figure showed that RF-EMF exposure decreased the total length of the neurite and the number of the branch point. **$p < 0.01$, and ***$p < 0.001$ by Student *t*-test.

SUPPLEMENTARY FIGURE S4

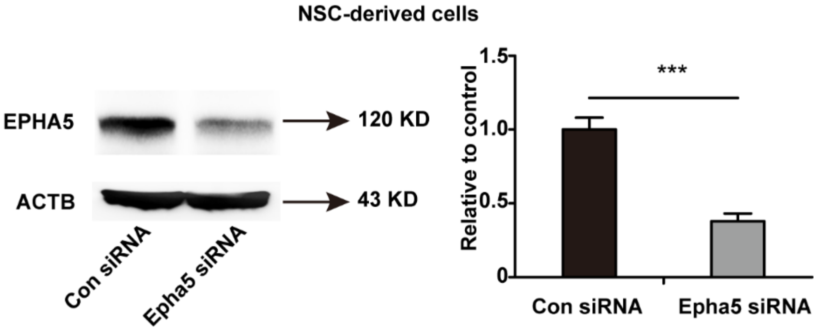

FIGURE S4 *Epha5* siRNA treatment remarkably decreased its protein expression. Data showed the representative western blot band and the statistical results. ***$p < 0.001$ by Student *t*-test.

SUPPLEMENTARY FIGURE S5

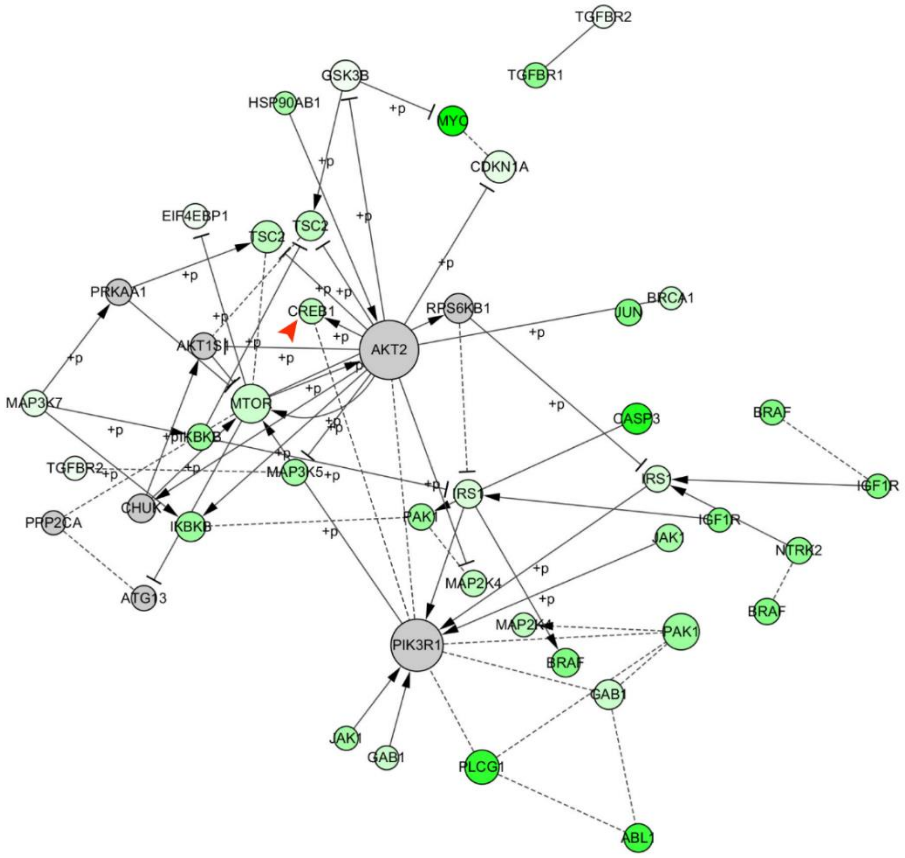

FIGURE S5 Phosphoprotein profile of key signaling pathways after RF-EMF exposure. The Core Signal-net showed the interactions of crucial factors in different signaling pathways based on the ratio of protein phosphorylation. The circle represents factors detected in CSP100^{plus} array. Arrow targets, activates downstream factor. "T", inhibits downstream factor. +p, promotes phosphorylation; -p, inhibits phosphorylation. The solid line represents direct regulation, while the dashed line represents indirect regulation.

SUPPLEMENTARY FIGURE S6

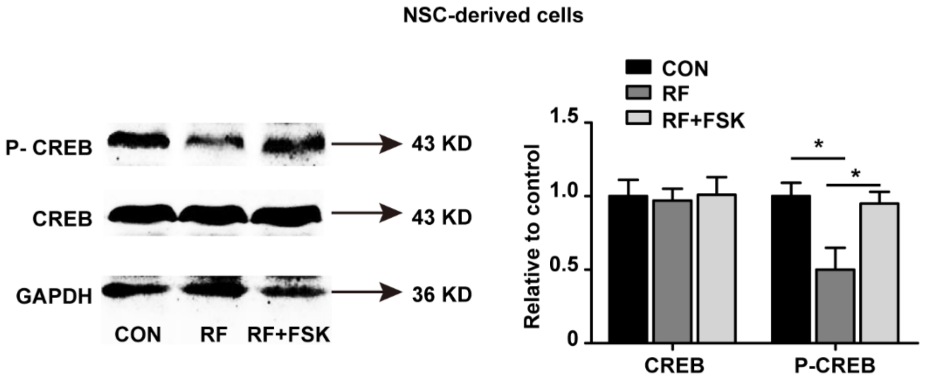

FIGURE S6 FSK treatment restored CREB phosphorylation in RF-EMF exposed NSCs. NSCs were induced differentiation under 4 W/kg RF-EMF exposure and 10 μM FSK treatment, the phosphorylation of CREB was then detected. *$p < 0.05$, one-way ANOVA followed by Boferroni post-tests.
