# Supplementary material for: 1800 MHz Radiofrequency Electromagnetic Field Impairs Neurite Outgrowth Through Inhibiting EPHA5 Signaling
Source: Front Cell Dev Biol. 2021 Apr 12;9:657623. doi: 10.3389/fcell.2021.657623 (PMC8075058; doi:10.3389/fcell.2021.657623)

Figure 3 panel (e)

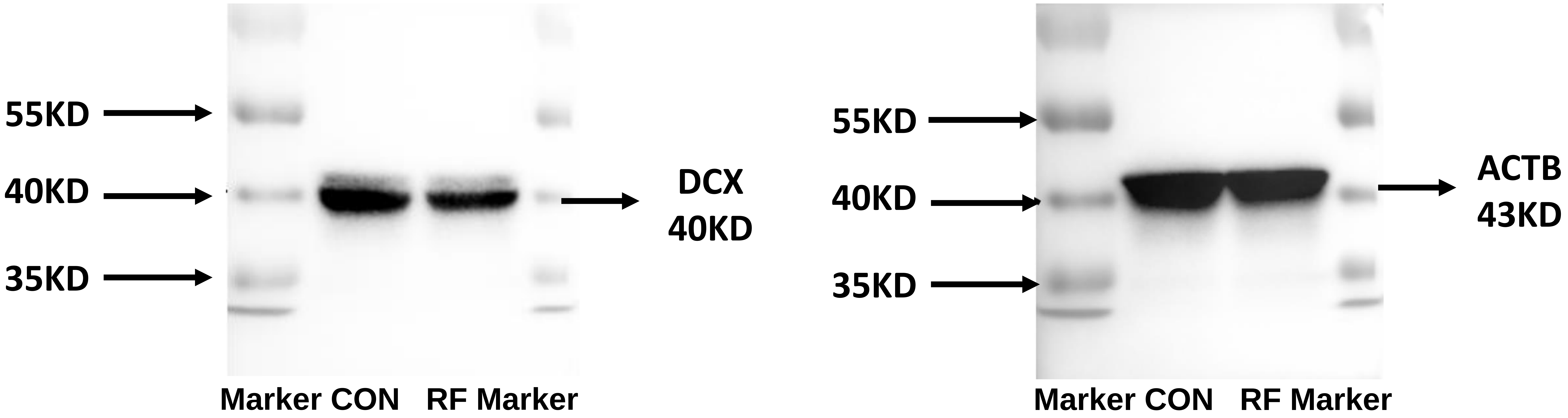

Figure 4 panel (c)

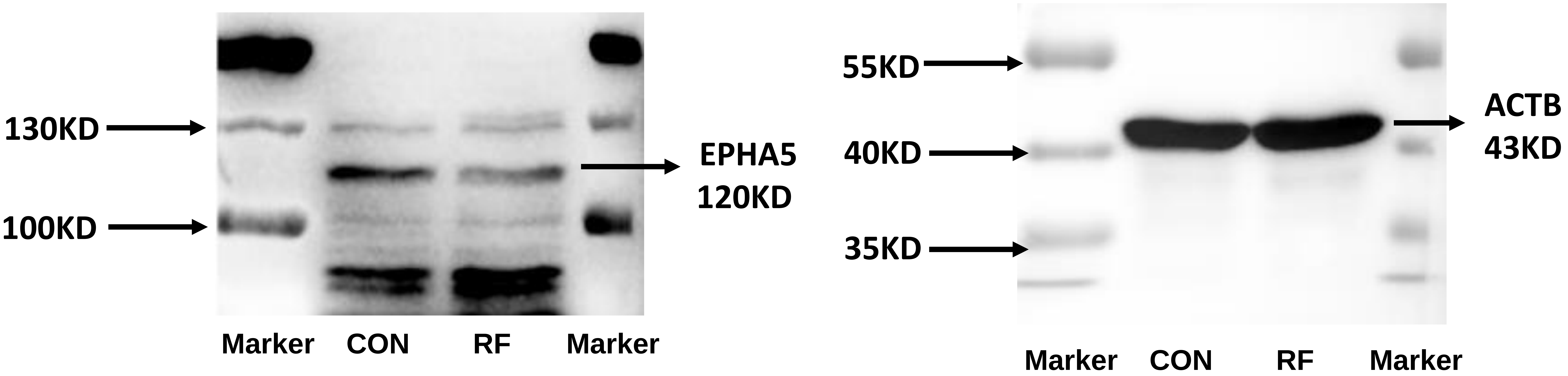

Figure 4 panel (d)

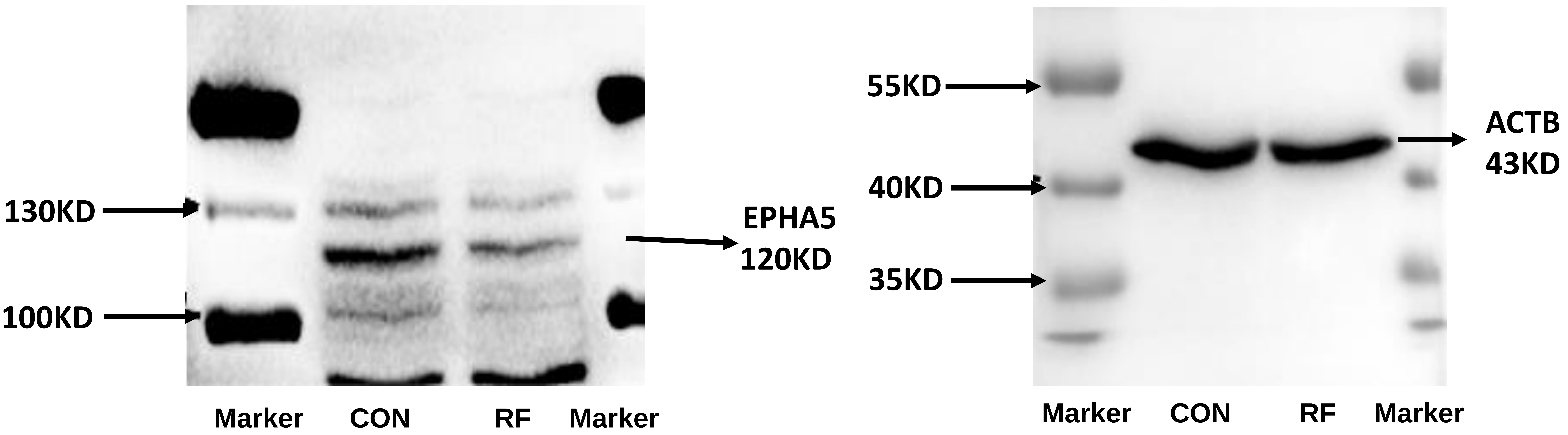

Figure 5 panel (g)

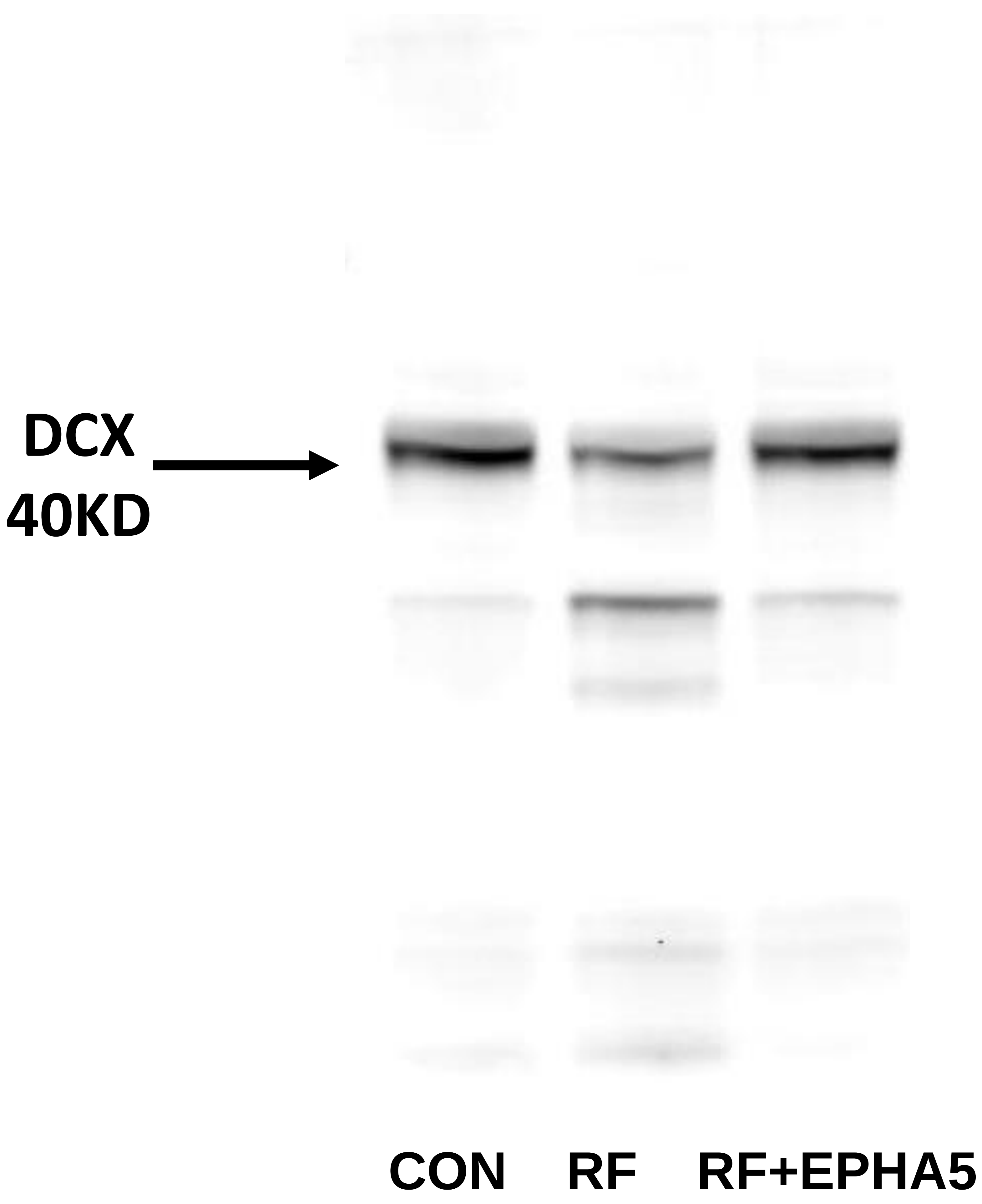

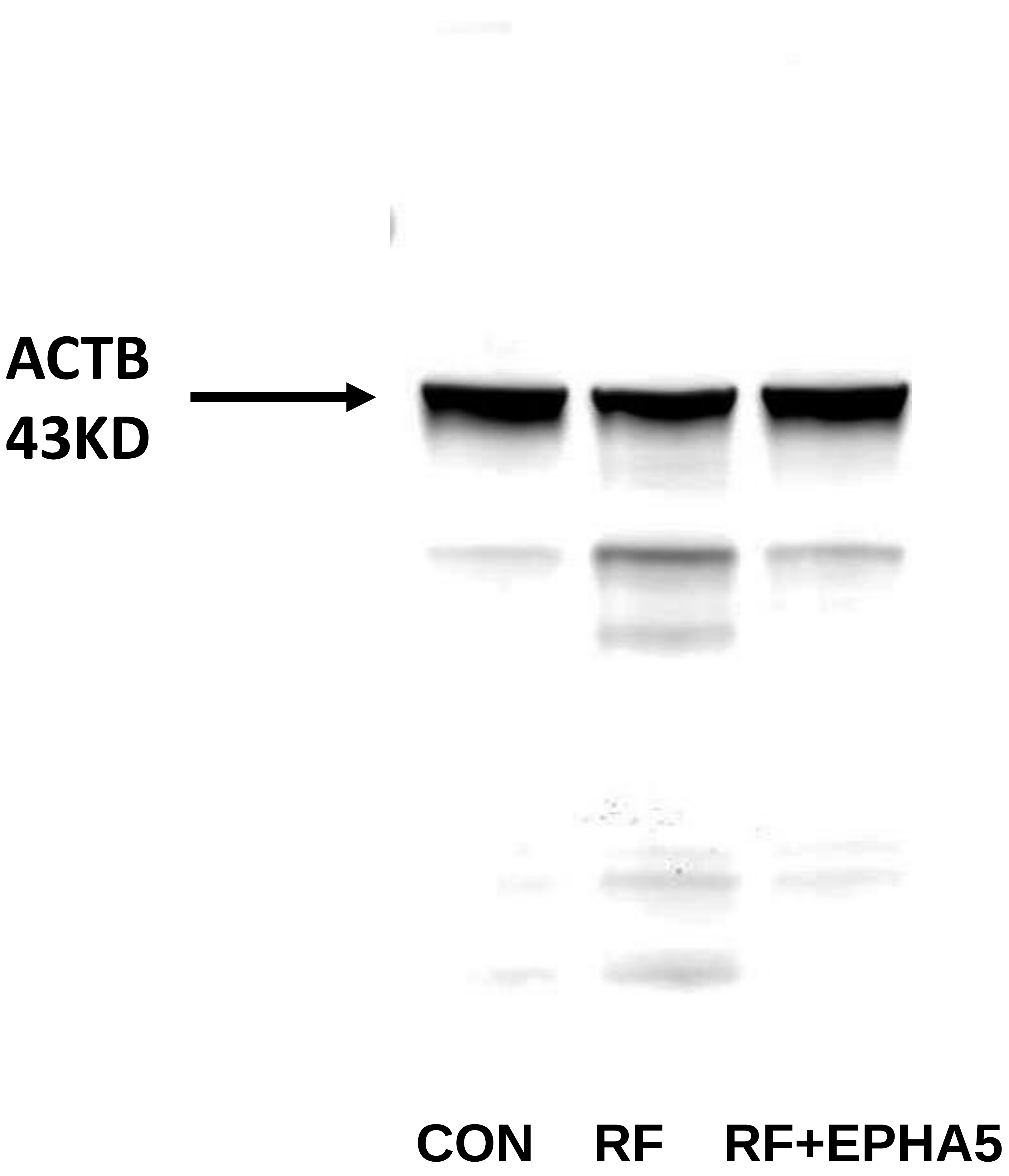

Figure 6 panel (a)

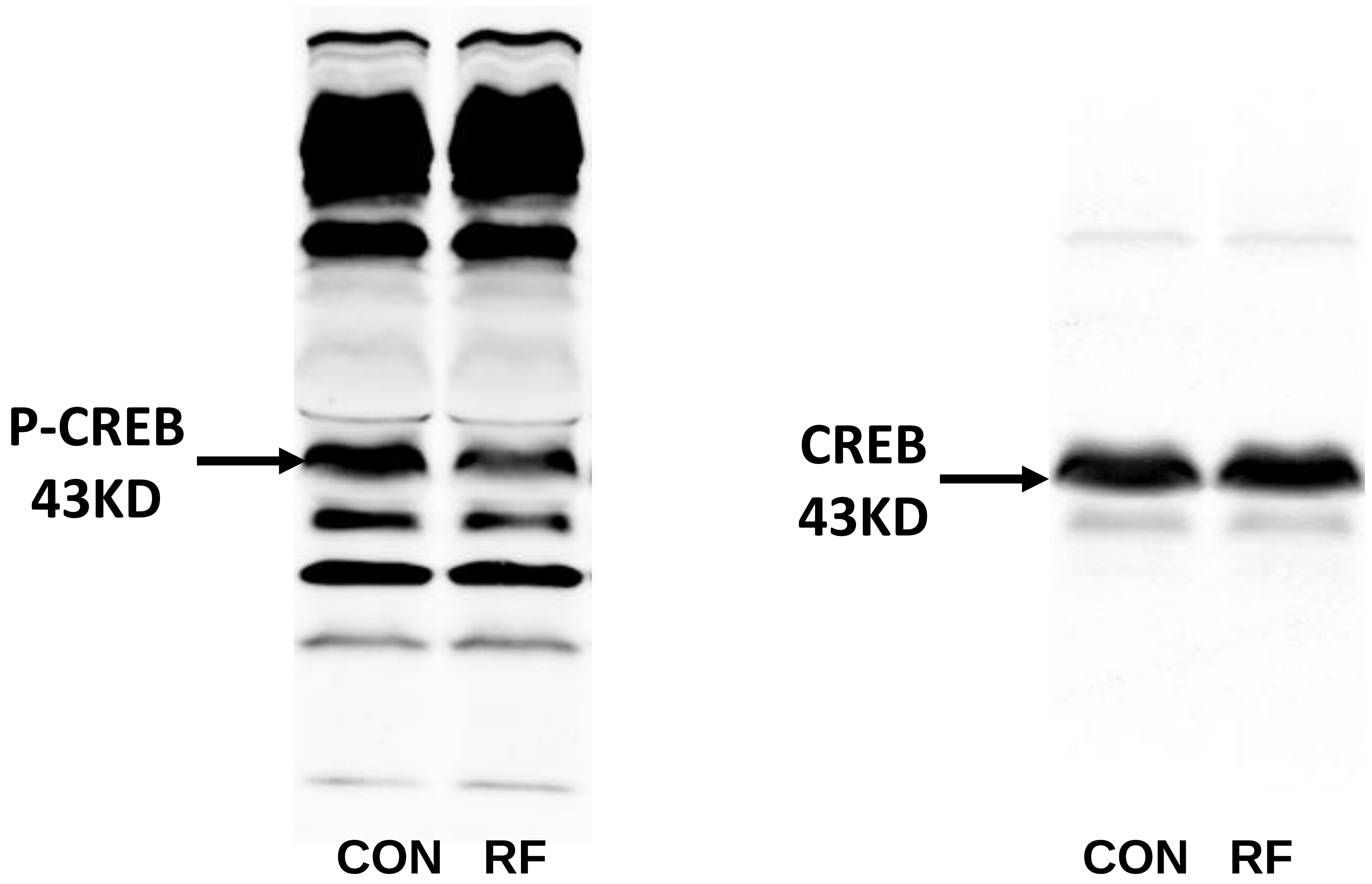

Figure 6 panel (b)

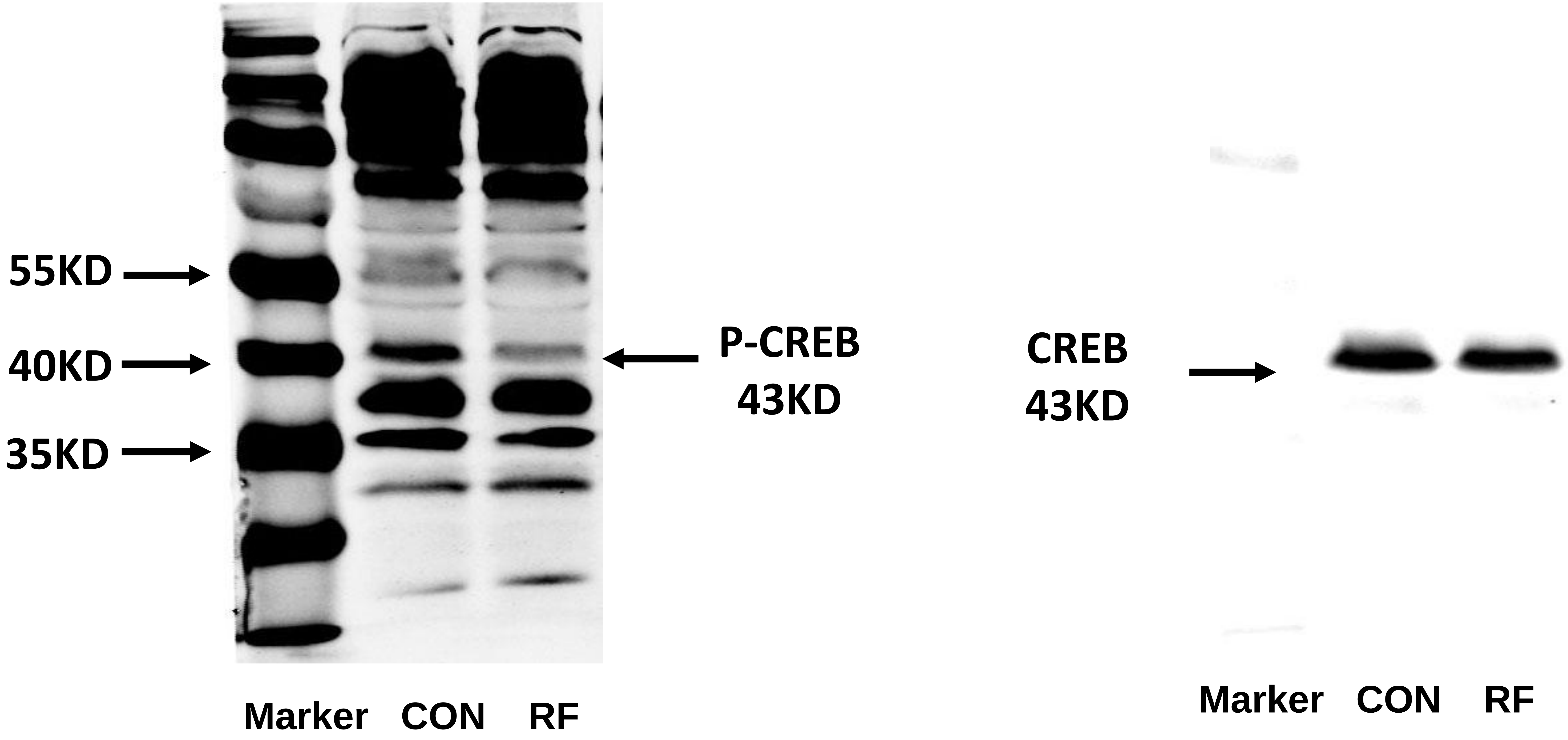

Figure 6 panel (c)

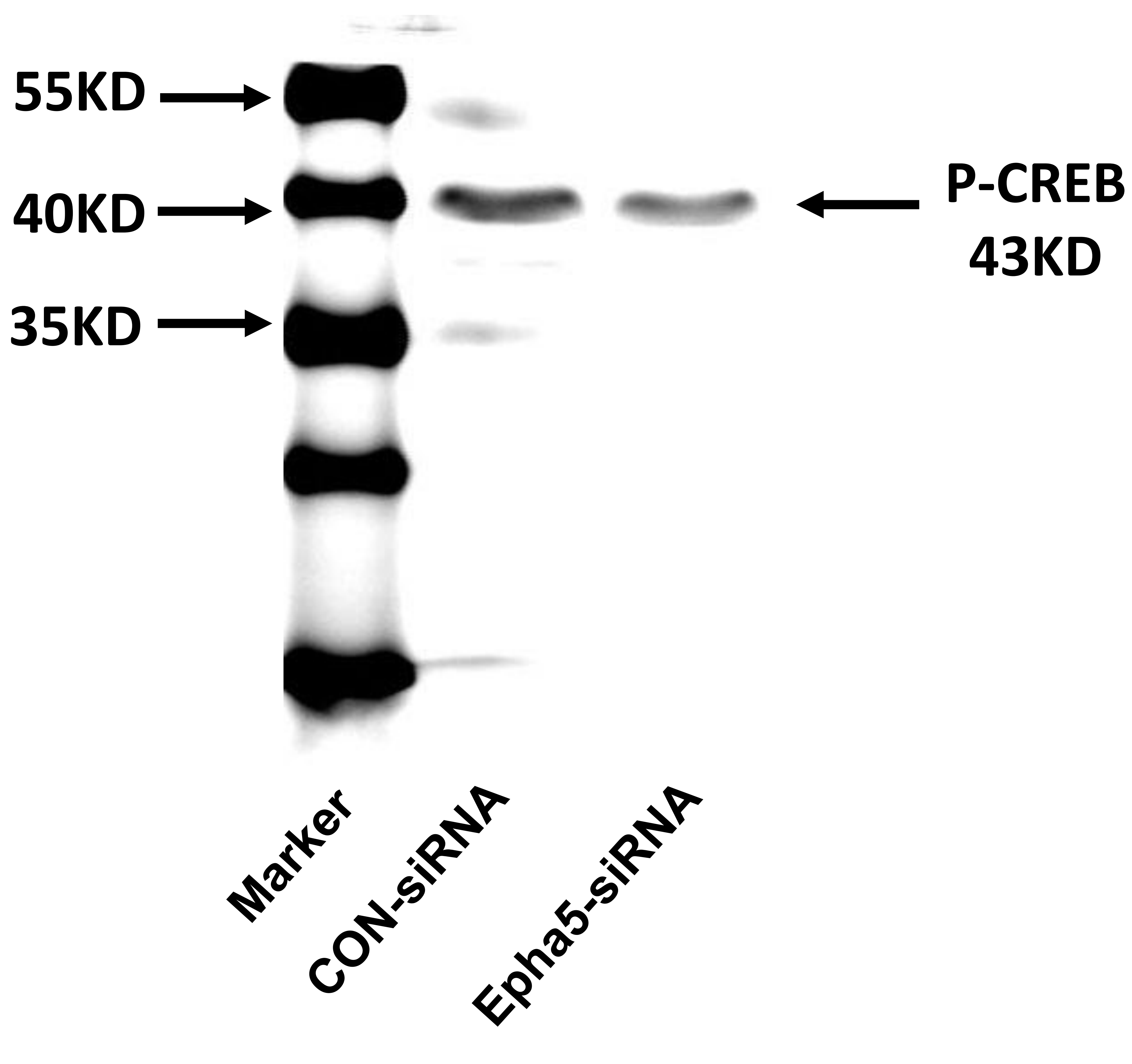

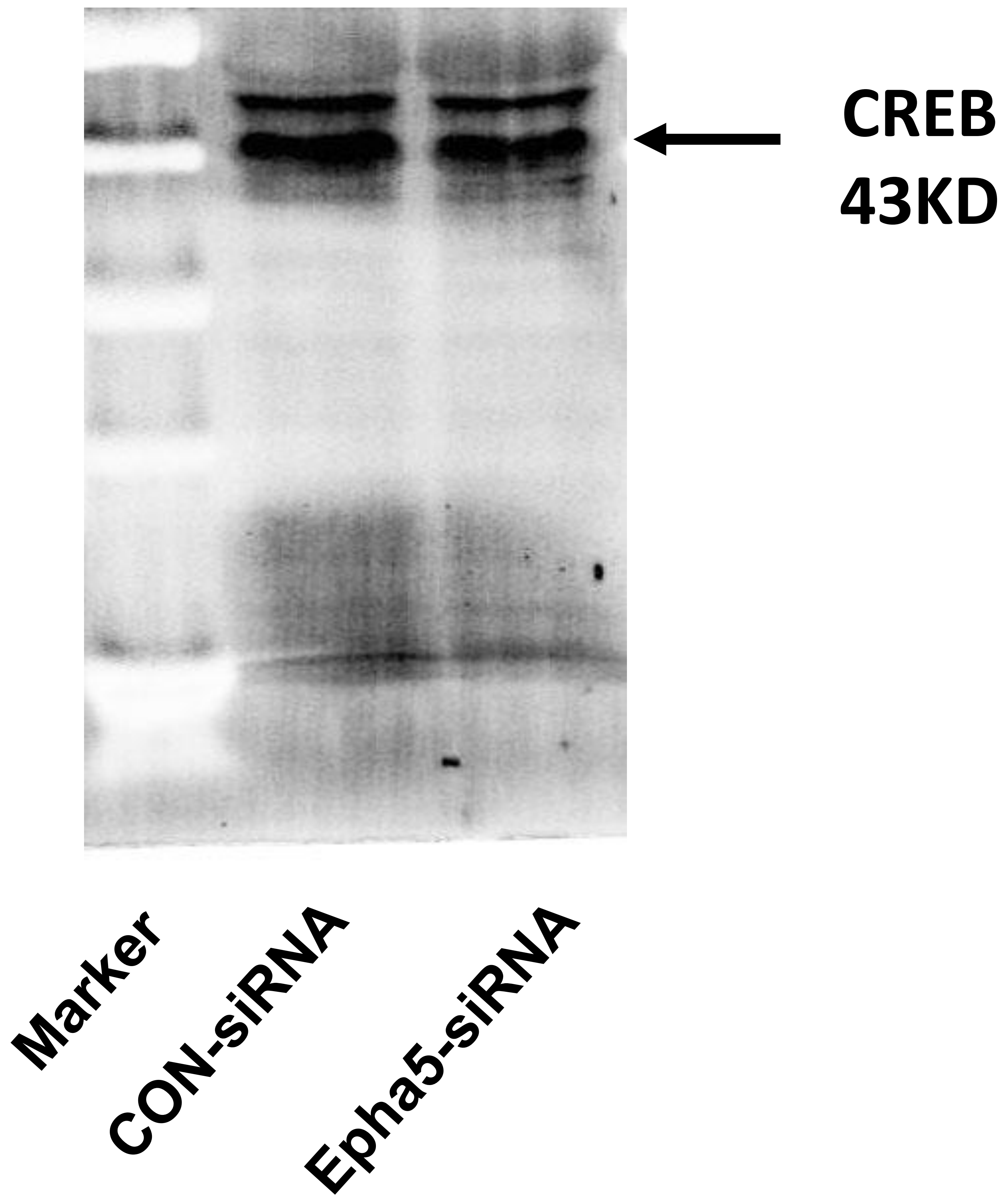

Figure 6 panel (d)

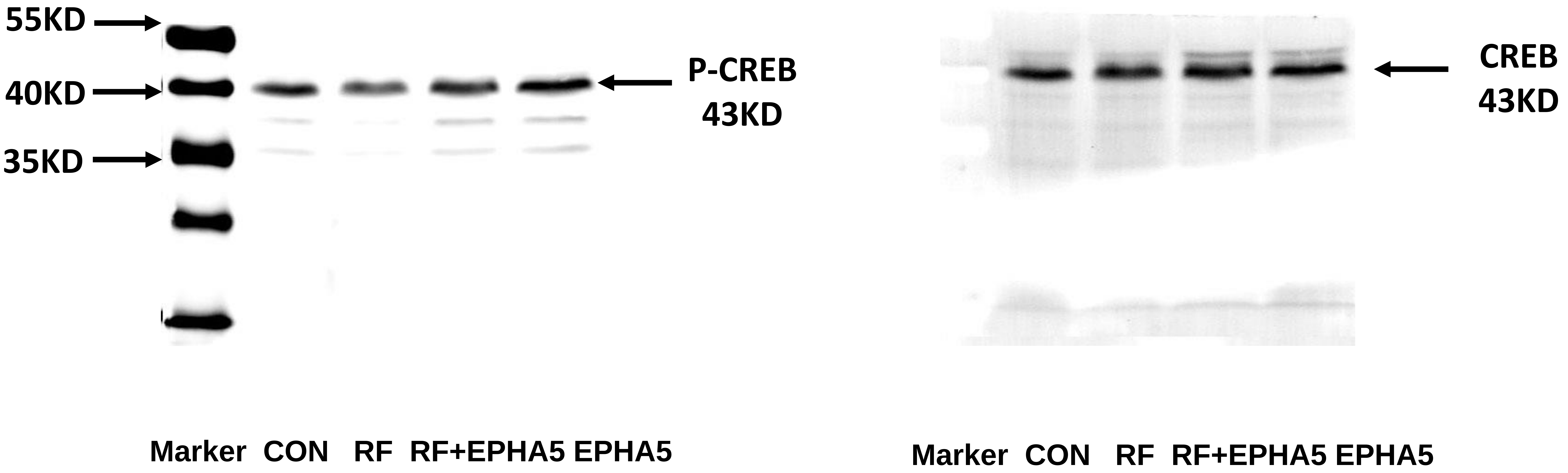

Figure 6 panel (e)

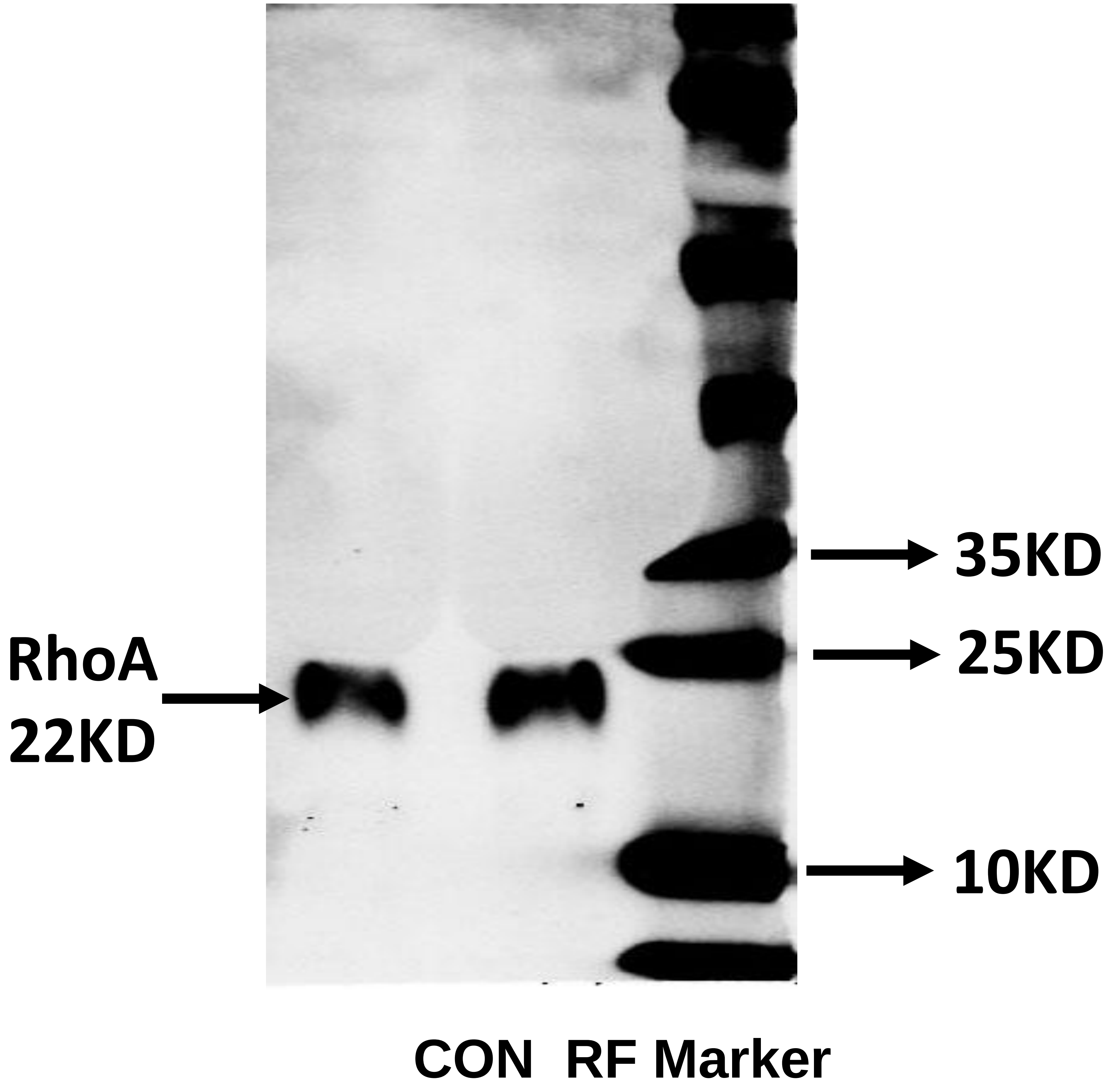

Figure 6 panel (f)

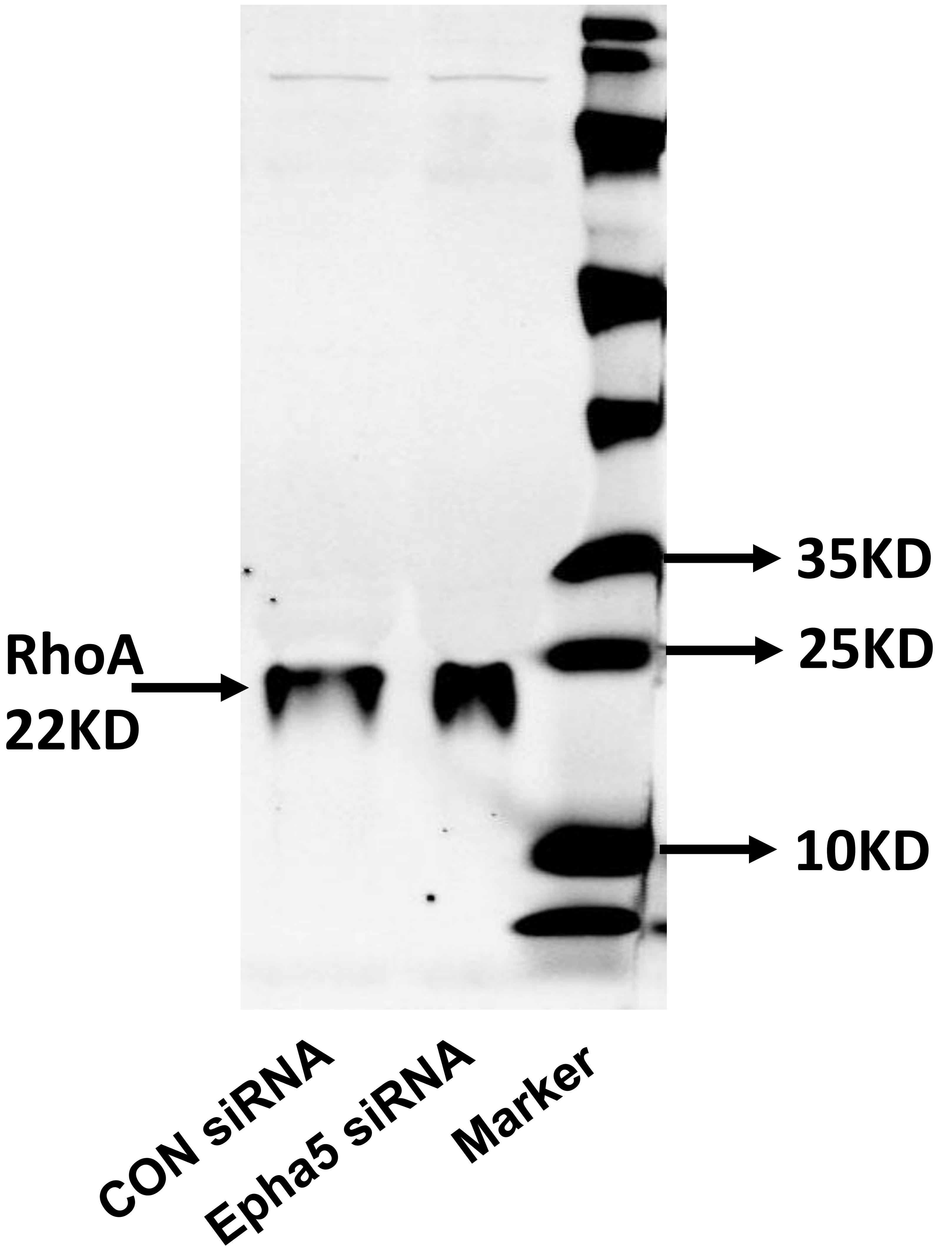

Figure 6 panel (g)

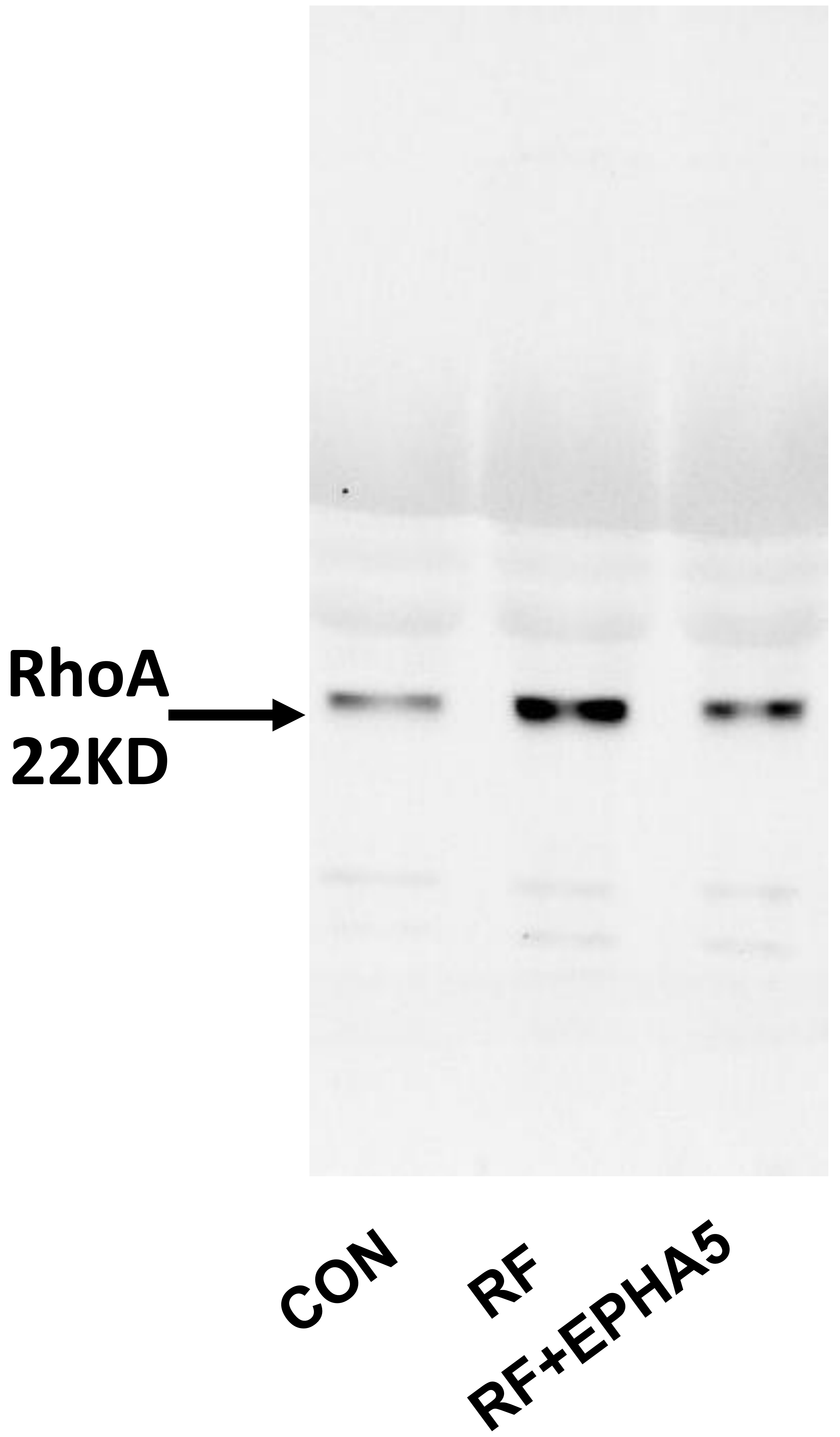

Figure 7 panel (e)

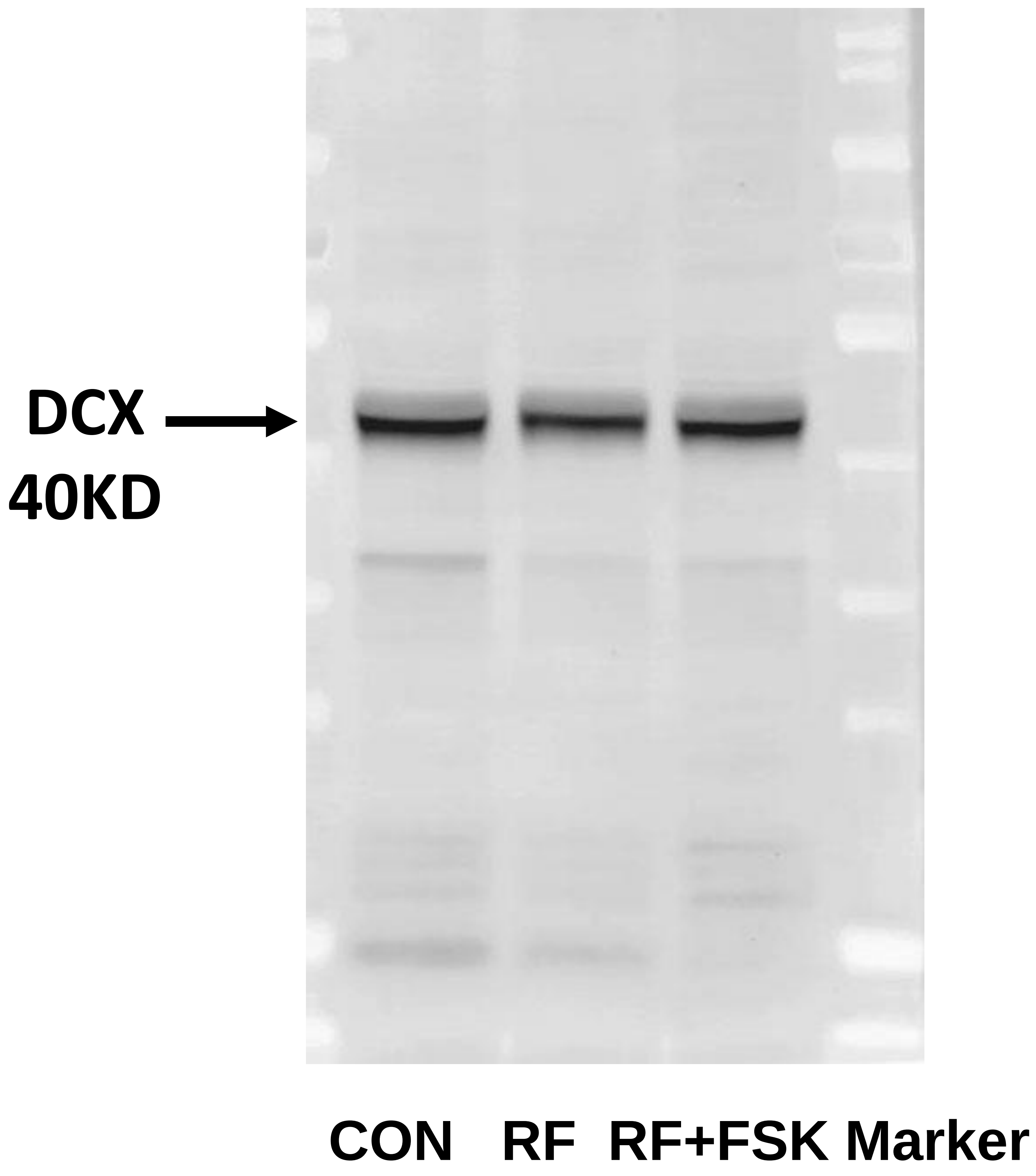

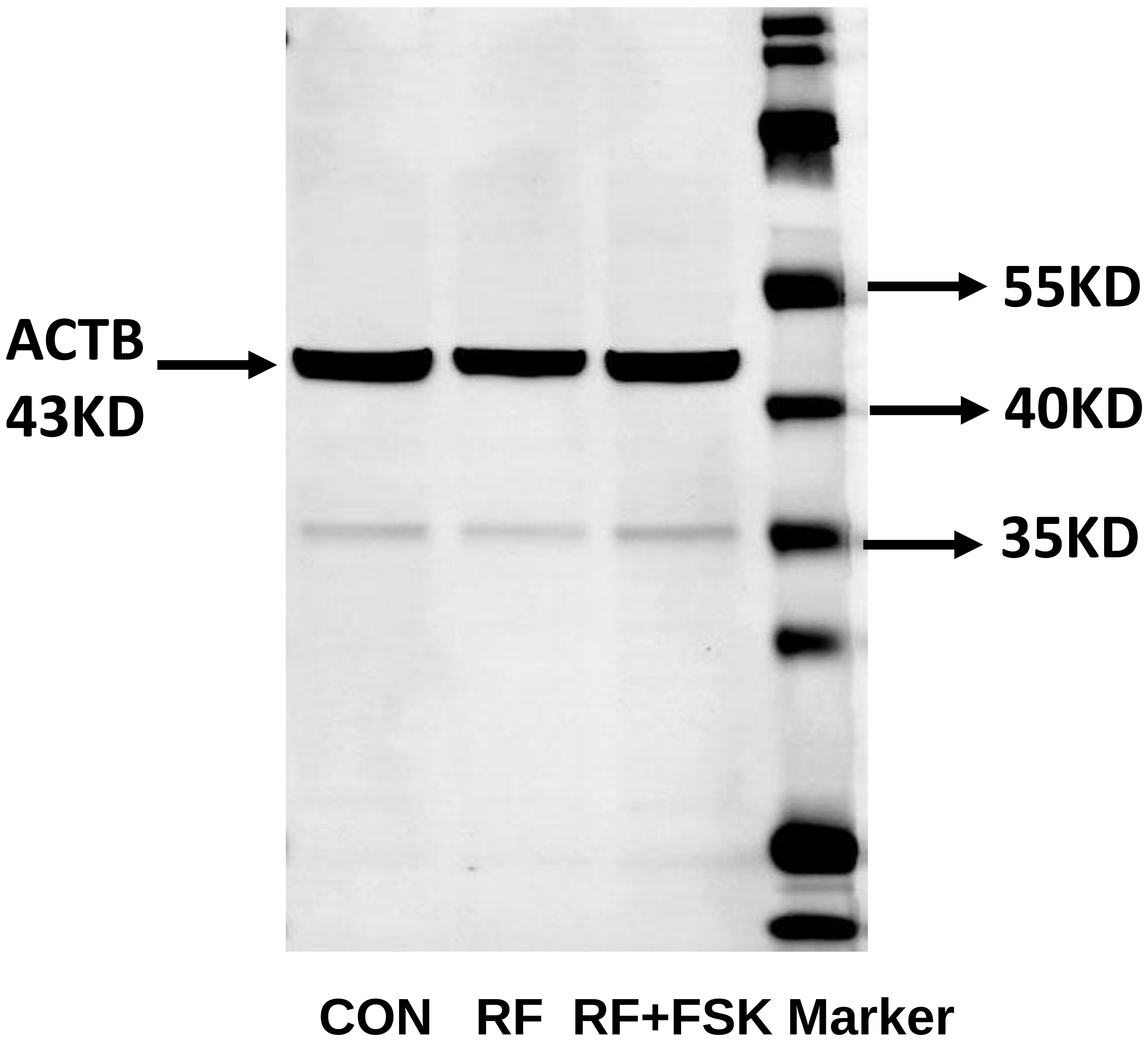

Figure 7 panel (g)

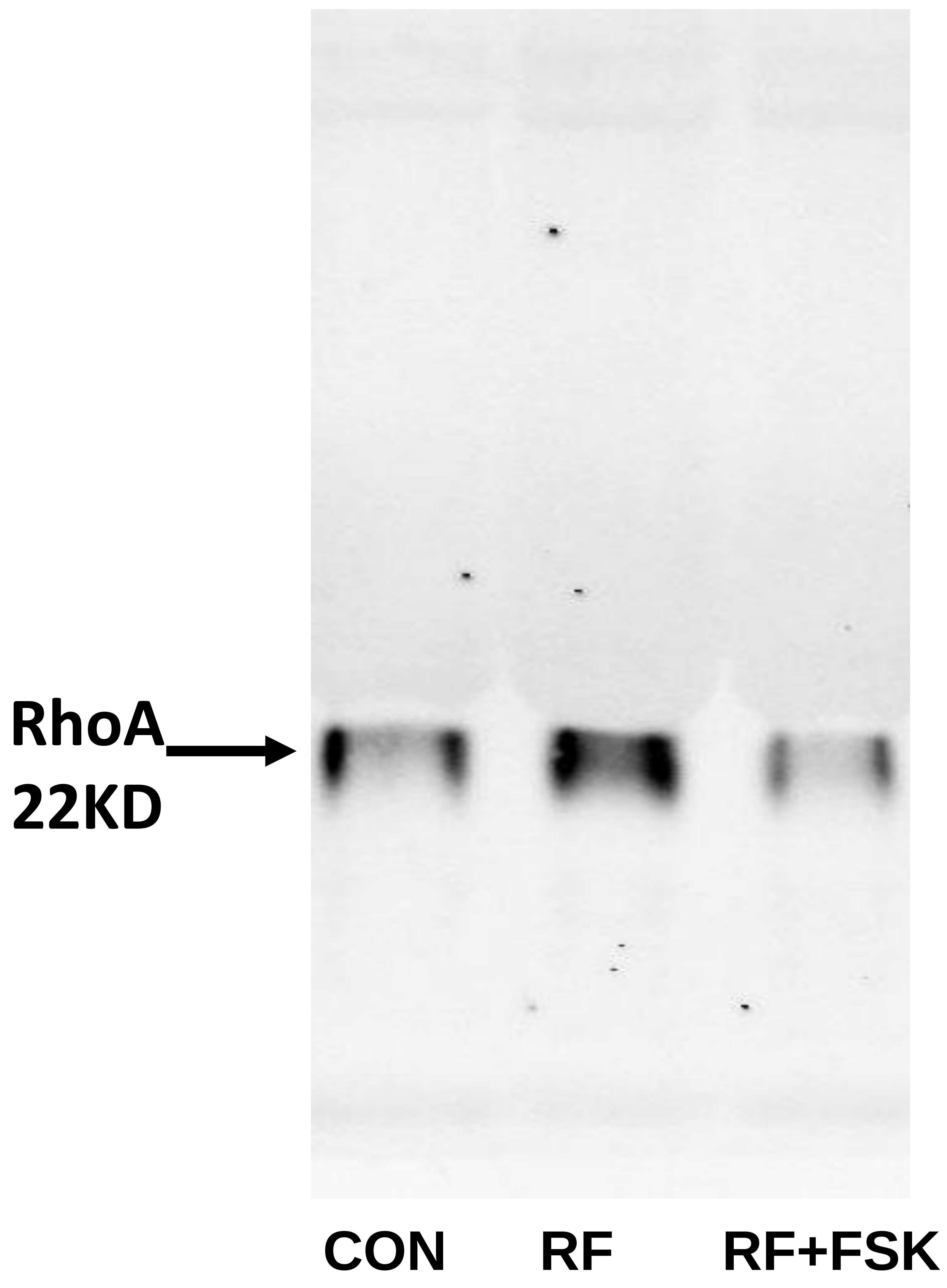

Figure S1

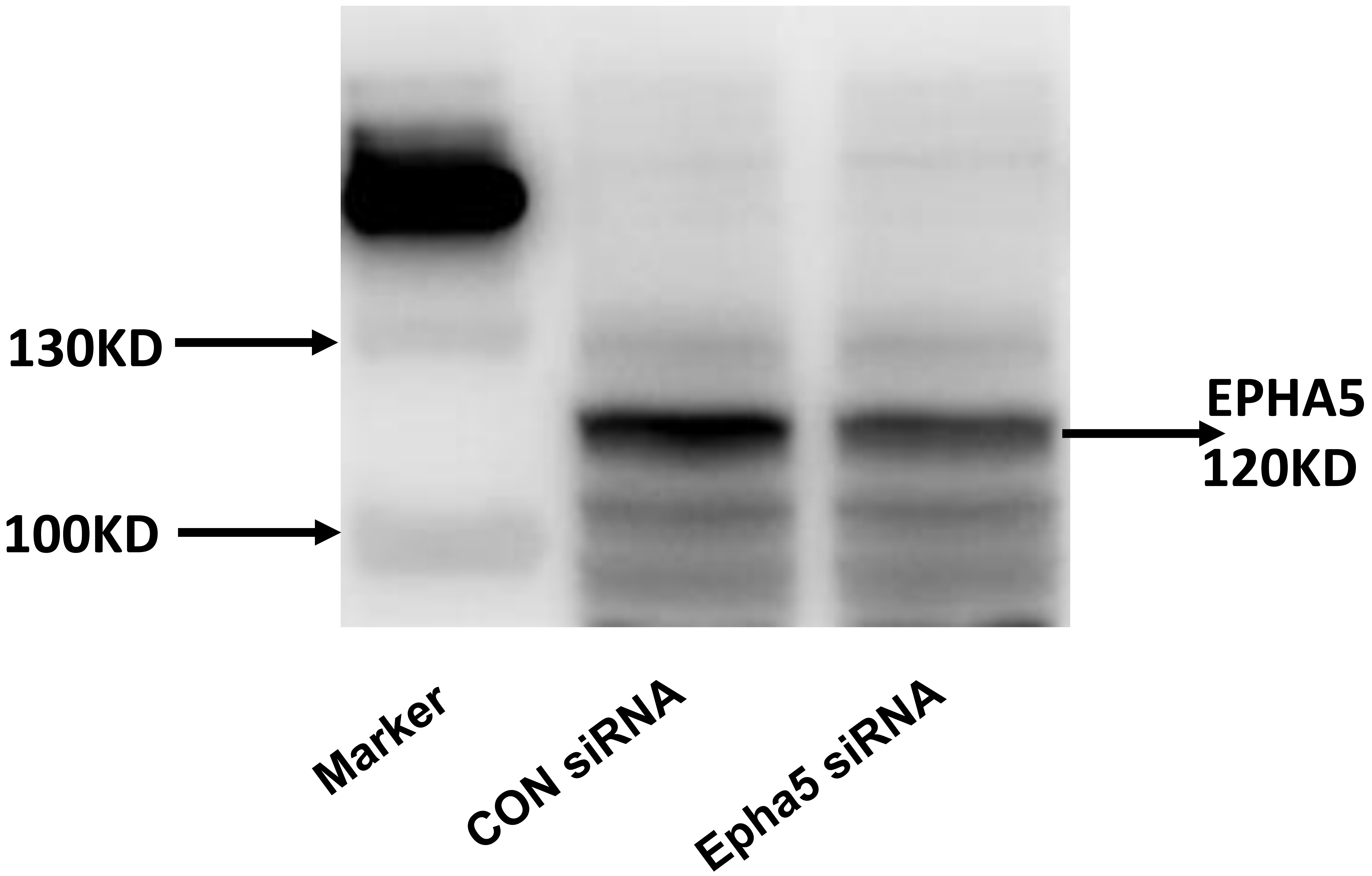
130KD
100KD
EPHA5
120KD
Marker
CON siRNA
Epha5 siRNA

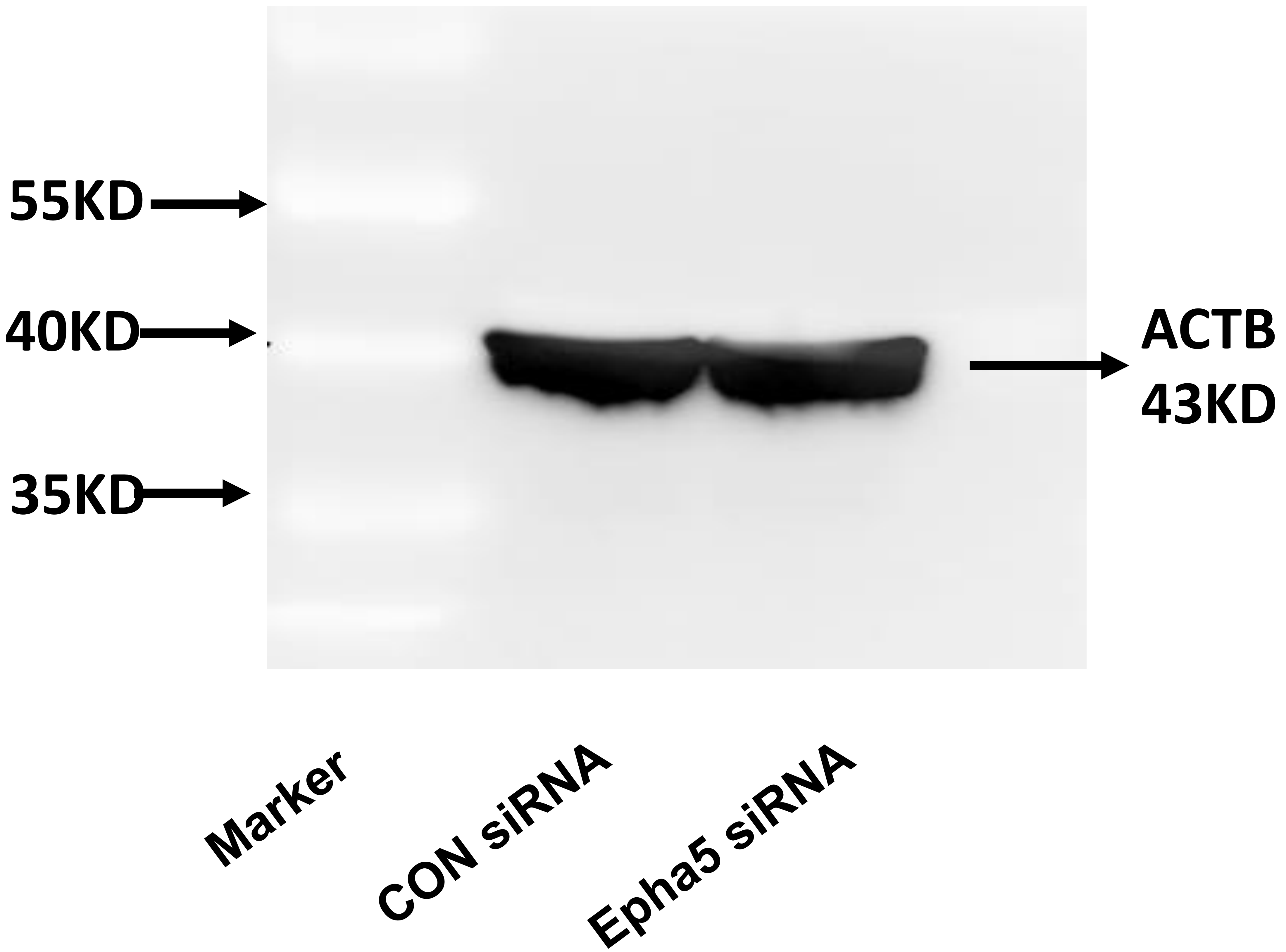
55KD
40KD
35KD
ACTB
43KD
Marker
CON siRNA
Epha5 siRNA

Figure S3

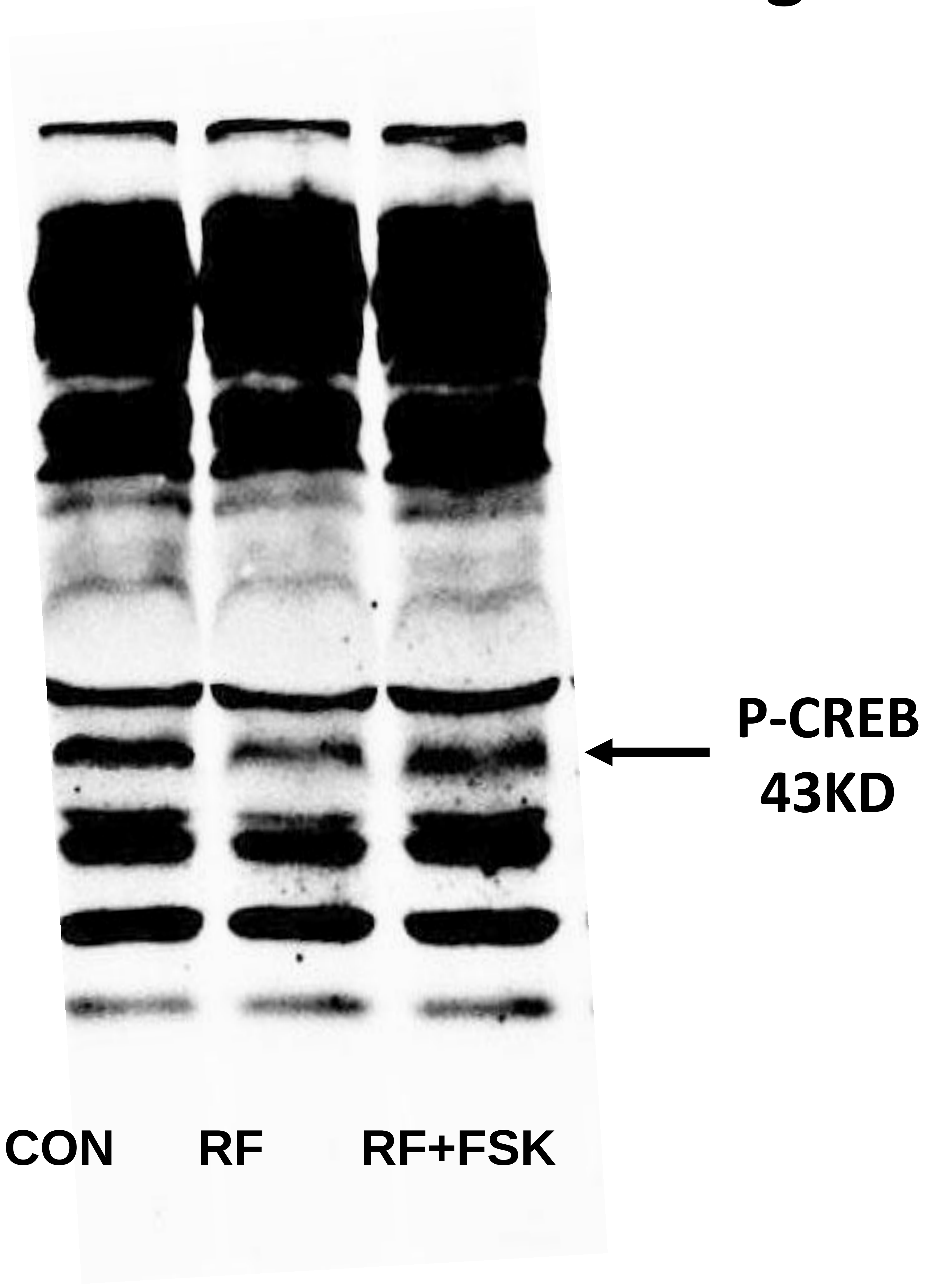

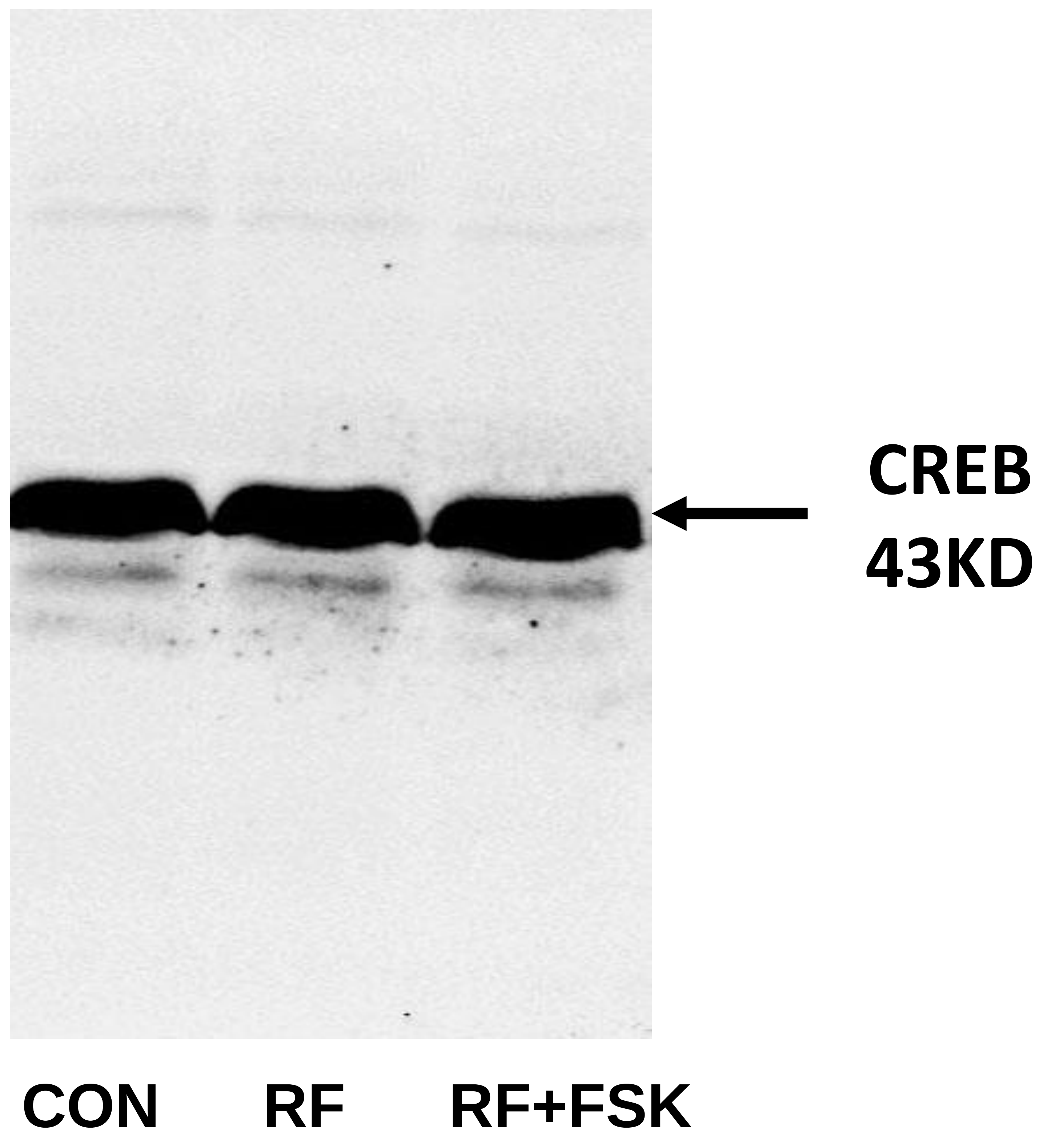

Supplement: Supplementary file 2 [file Data_Sheet_2.PDF]
